# γ-Aminobutyric Acid Type A (GABA_A_) Receptor Subunits Play a Direct Structural Role in Synaptic Contact Formation via Their N-terminal Extracellular Domains*

**DOI:** 10.1074/jbc.M116.714790

**Published:** 2016-04-20

**Authors:** Laura E. Brown, Martin W. Nicholson, Jessica E. Arama, Audrey Mercer, Alex M. Thomson, Jasmina N. Jovanovic

**Affiliations:** From the Research Department of Pharmacology, UCL School of Pharmacy, University College London, London WC1N 1AX, United Kingdom

**Keywords:** cell culture, γ-aminobutyric acid (GABA), inhibition mechanism, protein domain, synapse

## Abstract

The establishment of cell-cell contacts between presynaptic GABAergic neurons and their postsynaptic targets initiates the process of GABAergic synapse formation. GABA_A_ receptors (GABA_A_Rs), the main postsynaptic receptors for GABA, have been recently demonstrated to act as synaptogenic proteins that can single-handedly induce the formation and functional maturation of inhibitory synapses. To establish how the subunit composition of GABA_A_Rs influences their ability to induce synaptogenesis, a co-culture model system incorporating GABAergic medium spiny neurons and the HEK293 cells, stably expressing different combinations of receptor subunits, was developed. Analyses of HEK293 cell innervation by medium spiny neuron axons using immunocytochemistry, activity-dependent labeling, and electrophysiology have indicated that the γ2 subunit is required for the formation of active synapses and that its effects are influenced by the type of α/β subunits incorporated into the functional receptor. To further characterize this process, the large N-terminal extracellular domains (ECDs) of α1, α2, β2, and γ2 subunits were purified using the baculovirus/Sf9 cell system. When these proteins were applied to the co-cultures of MSNs and α1/β2/γ2-expressing HEK293 cells, the α1, β2, or γ2 ECD each caused a significant reduction in contact formation, in contrast to the α2 ECD, which had no effect. Together, our experiments indicate that the structural role of GABA_A_Rs in synaptic contact formation is determined by their subunit composition, with the N-terminal ECDs of each of the subunits directly participating in interactions between the presynaptic and postsynaptic elements, suggesting the these interactions are multivalent and specific.

## Introduction

GABA_A_ receptors (GABA_A_Rs)^3^ represent a large and diverse family of Cl/HCO_3_-permeable ion channels that mediate synaptic inhibition at the majority of inhibitory synapses in the brain (1–4). As such, GABA_A_R are essential for normal functioning of the brain, and their malfunction has been directly linked to a number of neurological and psychiatric disorders (5–7).

The structural diversity of these receptors has long been recognized as a key factor in determining the range of functional and pharmacological properties they display. Native GABA_A_Rs are hetero-pentamers of subunits that have multiple isoforms and are classified as follows: α(1–6), β(1–3), γ(1–3), δ, ϵ, π, and θ (2), with a common transmembrane topology comprising a large N-terminal extracellular domain, four transmembrane domains, and a major intracellular domain between transmembrane domains 3 and 4 (4, 8). The β3 and γ2 subunits are essential for synaptic inhibition and organism survival because mice bearing genetic deletion of these subunits die after birth (9, 10), while the individual isoforms of α subunit are associated with specific functions, such as anxiety, sedation, arousal, and others (11–13).

Synaptic GABA_A_Rs contain a γ2 subunit, two β subunits (β2 or β3), and two α subunits (α1, α2, α3 or α5) (14, 15). Although the β-subunits play a role in subcellular localization of GABA_A_Rs to axons, dendrites, or soma and insertion into the plasma membrane (16, 17), the presence of the γ2 subunit determines their localization to synaptic contacts. Different isoforms of α subunits appear, however, to be expressed more selectively at specific types of inhibitory synapses (3, 18, 19).

GABAergic synapse development is a precisely coordinated process that allows selective association between certain types of inhibitory axons and their postsynaptic targets, including the class of GABA_A_Rs that are clustered at the postsynaptic membrane (18, 20–22). This raises a question as to how the pre- and post-synaptic partners are recognized during the initiation of synaptic contacts. This question remains largely unanswered due to the complexity and bi-directional nature of trans-synaptic signaling between the pre- and post-synaptic elements *in vivo* (23–26). However, various *in vitro* heterologous co-culture assays have been successfully applied to study these mechanisms and to test the role of individual molecules in synapse formation, revealing the role of adhesion proteins, such as NCAM and cadherins, and trans-synaptic protein complexes, such as those formed by neuroligins and neurexins (27–32). In addition, we have recently demonstrated that the GABA_A_Rs themselves act as synaptogenic proteins that can induce the formation and functional maturation of inhibitory synapses using a co-culture model system incorporating the GABAergic MSNs and HEK293 cells expressing these receptors at the cell surface (33). These synapses are stable and show the ultrastructural characteristics typical of active synapses, and in functional experiments, they support spontaneous and action potential-driven postsynaptic GABAergic currents. This indicates that GABA_A_Rs participate in the formation of inhibitory synapses as structural proteins in addition to being the essential functional components that mediate synaptic inhibition as GABA-gated ion channels.

Specific localization of different classes of GABA_A_ receptors to distinct inhibitory synapses was also observed in the striatum and globus pallidus of the basal ganglia (34–36). These regions are primarily (∼95%) populated by GABAergic medium spiny neurons (MSNs) (37), the main projection neurons that form direct output pathways to the brainstem, to control motor function, and to the thalamus and cortex, to regulate behavior, emotions, and cognition (38, 39). MSNs form a finely tuned network of inhibitory connections within and between the striatum and globus pallidus (40) with α2/β3/γ2-GABA_A_ receptors being predominantly expressed in the former and α1/β2/γ2-GABA_A_ receptors in the latter region (34). Although striatal MSNs themselves are predominantly innervated by striatal GABAergic interneurons, their axonal projections target the MSNs in the globus pallidus and form synapses which incorporate predominantly the α1/β2/γ2-GABA_A_Rs. Similarly, the pallidal MSNs form synapses that target neurons outside of the basal ganglia, which also predominantly incorporate the α1/β2/γ2-GABA_A_ receptors (35, 41). These data collectively identify the α1/β2/γ2- and α2/β3/γ2-GABA_A_Rs as the most abundant and functionally important receptor subtypes in the basal ganglia.

To investigate further the structural role of GABA_A_R in synapse formation, we have generated new HEK293 cell lines stably expressing specific subunit combinations that were subsequently co-cultured with striatal MSNs. Analyses of the innervation of these cells by MSN axons have indicated that the presence of the γ2 subunit is necessary but not sufficient for a rapid formation of active synaptic contacts. The “synaptogenic” effects of this subunit are influenced by the type of α and β subunits present in the receptor pentamer, with the α1/β2/γ2-GABA_A_ receptor representing the most potent combination and the α2/β3/γ2-GABA_A_ receptor showing very little or no activity. Our experiments have also indicated that the large N-terminal ECDs of GABA_A_R subunits are directly involved in contact formation. Although the presynaptic binding partners of GABA_A_Rs remain to be identified, our results suggest that multiple interactions involving all of the subunits incorporated into the receptor pentamer are likely to contribute to the formation of GABAergic synapses.

## Experimental Procedures

### 

#### 

##### Primary Neuronal Cultures

Timed-pregnant BALB/c mice (Harlan, UK; the number of pregnant females used was ∼30) were housed and sacrificed according to United Kingdom Home Office guidelines (and European Communities Council Directive of 24 November, 1986 (86/609/EEC)). The project was formally approved by the UCL School of Pharmacy Ethics Committee.

Primary cultures of MSNs were prepared as described previously (42, 43). Striata were dissected from embryonic day 16–17 (E16–17) mouse embryos, dissociated by trituration in Ca^2+^- and Mg^2+^-free HEPES-buffered saline solution (HBSS; catalog no. 14180-046, Gibco), and plated at a density of 60,000 cells per well in Neurobasal medium (catalog no. 21103-049, Gibco) containing B27 supplement (catalog no. 17504-044, Gibco), glutamine (2 mm; catalog no. 25030-024, Gibco), penicillin (50 units/ml; catalog no. 15070-63, Gibco), streptomycin (50 μg/ml; 15070-063, Gibco), and glucose (6 mm; catalog no. G8769, Sigma) on poly-l-lysine- (0.1 mg/ml; catalog no. P6282, Sigma), and laminin-coated (0.01 mg/ml; catalog no. L2020, Sigma) glass coverslips (13 mm in diameter; catalog no. 631-0150, VWR International). Cultures were maintained in a humidified 37 °C/5% CO_2_ incubator for 14 days prior to experimentation.

##### Preparation of Stable HEK293 Cell Lines

HEK293 cells (2 × 10^6^) were transfected using Lipofectamine LTX (catalog no. 15338-100, Invitrogen) with different mouse GABA_A_R subunit cDNAs as follows: α1 or α2 pcDNA3.1^(+)^, incorporating the G418 disulfate (neomycin; catalog no. G5013, Sigma) resistance gene and β2 or β3 pcDNA3.1^(+)^, incorporating the Zeocin resistance gene (catalog no. R25001 Gibco). Briefly, Opti-MEM I (500 μl, 11058-021 Gibco) was combined with each α/β cDNA construct (7.5 μg per construct) and Plus Reagent (15 μl, 15338-100 Invitrogen) and incubated at room temperature for 5 min. Subsequently, Lipofectamine LTX (8.75 μl, catalog no. 15338-100, Invitrogen) was added to the reaction mixture and left at room temperature for 30 min. After the addition of cell culture medium (3 ml), the content of the transfection reaction was added to the cells and incubated for 48 h at 37 °C. Cells were washed with PBS and re-plated at the ratios of 1:3, 1:5, 1:7, 1:10, 1:15, and 1:20, into new sterile 10-cm culture dishes for 24 h. Cells were selected with G418 (800 μg/ml; neomycin; catalog no. G5013, Sigma) and Zeocin antibiotics (800 μg/ml; catalog no. R25001, Gibco) and incubated at 37 °C until colonies formed. After 7 days, ∼5–20 single colonies were selected and gradually scaled up from a 24-well plate to a 6-well culture plate, a 60-mm plate, a 10-cm plate and finally to a cell culture (T-75) flask. Immunoblotting and immunocytochemistry were used to characterize the expression of GABA_A_R subunits.

##### Co-cultures of MSNs and HEK293 Cells

HEK293 cells were plated at a density of 3 × 10^5^ cells per well in a 6-well plate and the following day transiently transfected with mCherry alone (200 ng; mCherry-N1 Mammalian Expression Vector, catalog no. P632523, Invitrogen) or in combination with GABA_A_R γ2 subunit cDNA (200 ng plus 800 ng of γ2 pcDNA^TM^3.1^(+)^ Mammalian Expression Vector, catalog no. V870-20, Invitrogen) using Effectene reagent (catalog no. 301425, Qiagen). The efficiency of transient transfection with both mCherry and γ2 cDNA was 60–70%. Cells were trypsinized (catalog no. 25300-054, Gibco) 24 h post-transfection and added to cultures of MSNs at a density of 30,000 cells/well of a 24-well plate. Co-cultures were fixed after 24 h and synaptic contacts analyzed by immunolabeling and confocal microscopy. In experiments in which the activity of GABA_A_Rs was suppressed, bicuculline (25 μm; catalog no. 0130, Tocris Bioscience) diluted in DMSO, or the equivalent amount of DMSO (the final concentration below 1%), was added within 30 min of plating the HEK293 cells into the co-culture and incubated for 24 h.

##### Analysis of GABA_A_R Subunit Expression Using ELISA

To characterize the cell surface and total expression levels of GABA_A_Rs, HEK293 cells stably expressing different combinations of GABA_A_R subunits were seeded into sterile 24-well culture plates at a density of 75,000 cells per well. The following day, cells were transfected with mCherry-N1 mammalian expression vector (40 ng; catalog no. P632523, Invitrogen) plus empty pcDNA^TM^3.1^(+)^ mammalian expression vector (160 ng; catalog no. V870-20 Invitrogen; samples were labeled as α/β-HEK293) or mCherry-N1 mammalian expression vector (40 ng) plus γ2 pcDNA^TM^3.1^(+)^ mammalian expression vector (160 ng; samples labeled as α/β/γ-HEK293) using Effectene reagent (catalog no.301425, Qiagen) and incubated overnight. Following transfection, the cultures were washed with PBS, fixed using 4% paraformaldehyde (catalog no. 158127, Sigma), 4% sucrose (catalog no. 443815S, VWR)/PBS (PFA) for 10 min, and processed as described previously (43–45), using the mouse anti-β2/3 primary antibody (1 μg/ml; MAB34, bd17 clone, Merck Millipore) and the secondary anti-mouse IgG conjugated to horseradish peroxidase (HRP) (1:2500; catalog no. 31450, Invitrogen). Controls omitting the primary antibody were used to determine the background levels of peroxidase and nonspecific binding of secondary antibodies. The assay was determined to be linear within the range of 0.5–2 μg/ml of the primary antibody in the presence of excess secondary antibody. Values were expressed as mean ± S.D., with individual data points from α/β-HEK293 and α/β/γ-HEK293 samples paired within separate experiments. The statistical analysis was performed using the paired *t* test (GraphPad Prism 6.0), with *p* < 0.05 accepted as statistically significant.

##### FM4-64FX Uptake

To assess the functionality of synaptic contacts formed in co-culture, the lipophilic dye, FM4-64FX was used (catalog no. F34653, Molecular Probes). In these experiments, HEK293 cells were transfected with a green fluorescent protein (EGFP, pmaxGFP-Amaxa Lonza) instead of mCherry, to avoid cross-activation with FM4-64FX, and used to create the co-cultures as described above. After 23 h, cells were washed twice briefly with buffer A, pH 7.4, containing NaCl (149 mm), KCl (4 mm), CaCl_2_ (1.5 mm), MgCl_2_ (1.5 mm), glucose (10 mm), and HEPES (10 mm) before being incubated with FM4-64 FX (10 μm), diluted in buffer A, for 20 min at 37 °C. The FM4-64FX dye was aspirated, and cultures were washed twice briefly with cold buffer A, followed by two 10-min washes with ADVESEP-7 (500 μm; catalog no. A3723 Sigma) diluted in cold buffer A, at room temperature to remove excess dye. Finally, cells were washed twice briefly with cold buffer A, before fixation.

##### Immunocytochemistry

Cells were fixed with 4% PFA for 10 min, washed extensively, and incubated with bovine serum albumin (BSA; 1%, catalog no. A3294, Sigma) in PBS for 30 min to reduce any nonspecific binding of the primary antibodies. Cells were incubated overnight at 4 °C with the following primary antibodies directed against the N-terminal ECDs of the GABA_A_R subunits: rabbit anti-GABA_A_R α1 subunit (1:200 (46)); mouse anti-GABA_A_R β2/3 subunit (10 μg/ml; catalog no. MAB341, Merck Millipore, Billerica, MA); guinea pig anti-GABA_A_R α2 subunit (1:400; catalog no. 224104, Synaptic Systems); and guinea pig anti-GABA_A_Rγ2 subunit (1:2000 (34)). To detect intracellular proteins, the cells were washed, permeabilized with Triton X-100 (0.1%; catalog no. H514, Promega) in PBS for 30 min, and incubated with BSA/PBS (1%) for 30 min, to block nonspecific binding, followed by the addition of the rabbit anti-vesicular GABA transporter (VGAT) primary antibody (1:500; catalog no. 131013, Synaptic Systems) for 120 min. The binding of primary antibodies was subsequently visualized by binding of the appropriate goat anti-rabbit, anti-mouse, or anti-guinea pig secondary antibodies conjugated to AlexaFluor488 or AlexaFluor555 (1:750; catalog no. AP124S/A21422, Life Technologies, Inc.) or Cy5 (1:750; catalog no. AP1085, Merck Millipore) in BSA/PBS (1%) for 1 h. Cells were washed thoroughly and coverslips mounted using ProLong Gold antifade reagent (catalog no. P36930, Life Technologies, Inc.).

##### Confocal Microscopy and Quantification of Synaptic Contacts

Immunoreactivity was visualized using the laser scanning confocal microscope (Zeiss LSM 710 Meta, Germany) with ×40 or 63 oil-immersion objective. Light levels, detector gain, and offset were adjusted to avoid any saturation. Images were taken on a single plane of the cell or at different focal depths, with sequential z-stack sections taken at equal optimal intervals of 0.64 μm throughout the plane of the cell. Synaptic contacts were identified as regions of co-localization of the presynaptic marker VGAT or FM4-64FX dye, and the surface of the HEK293 cells was transfected with mCherry or EGFP and quantified using ImageJ (National Institutes of Health). Using the “Image Calculator” function followed by the “Analyze Particles” function, the percentages of partially co-localized pixels were quantified and summed throughout each plane of the z-stack. The data were analyzed statistically using Origin Pro 9.0 32 Bit software. The Gaussian distribution of the collected data was analyzed using the Shapiro-Wilk and Kolmogorov-Smirnov tests, and the non-parametric Mann-Whitney test was used to investigate statistical significance between different experimental groups.

##### Design, Expression, and Application of GABA_A_R N-terminal ECDs as Blocking Reagents

Polymerase chain reaction (PCR) was used to amplify the sequence of mouse α1, α2, β2, and γ2 N-terminal ECDs using their existing pRK5 vectors. Forward primers designed to the first 30 nucleotides (including the signaling peptide) and reverse primers corresponding to the last 30 nucleotides before the first predicted transmembrane domain of the mouse α1, α2, β2, and γ2 DNA sequences used were as follows: α1 forward, 5′-ATGAAGAAAAGTCGGGGTCTCTCTGACTA-3′, and reverse, 5′-AAAGTAGCCAATTTTTTCTCTTCAAGTGGAA-3′; α2 forward, 5′-ATGAAGACAAAATTGAGCACATGCAATGTAT-3′, and reverse, 5′-CCCAATTTTTCTTTTCAAGTGGAAATGAGC-3′; β2forward, 5′-ATGTGGAGAGTCCGGAAAAGGGGCTACTTT-3′, and reverse, 5′-GTAGCCAATGTTCCTTTTCAGCTTAAAGCT-3′; γ2 forward, 5′-ATGAGTTCGCCAAATACATGGAGCATTGGA-3′, and reverse, 5′-GCCCATTCTTCTGCTCAGATCGAAGTACAC-3′. Amplifications were performed using a three-step PCR involving Platinum *Pfx* DNA polymerase and 35 cycles of 94 °C for 2 min followed by a further 1 min, 65 °C for 1 min, and 68 °C for 1 min followed by a further 10 min. The baculovirus/Sf9 cell expression system was then used to express the N-terminal ECDs of the GABA_A_R α1, α2, β2, and γ2 subunits tagged with the His_6_ sequence at the C terminus according to the Invitrogen User Manual (catalog no. A10605 Bac-to-Bac TOPO cloning kit/catalog no. A10606 Bac-to-Bac TOPO expression system). Purification of ECDs from the virus-infected Sf9 cells (200 ml of culture pellets) was carried out under sterile conditions. The Sf9 cells were lysed with the buffer containing NaH_2_PO_4_ (20 mm, pH 8.0; catalog no. S9638 Sigma), Nonidet P-40 (0.5%; Ab14222, Abcam), and protease inhibitors mixture (5 μg/μl; catalog no. 05892791001, Roche Applied Science) and centrifuged at 11,180 × *g* for 15 min at 4 °C. The supernatant was subsequently removed, and the pellet was resuspended in the solubilization buffer containing NaH_2_PO_4_ (50 mm, pH 8.0), NaCl (150 mm), Nonidet P-40 (2%), deoxycholate (1%, 89904, ThermoScientific), and protease inhibitors (5 μg/μl) with rotation for 90 min at 4 °C. Solubilized proteins were dialyzed in dialysis buffer containing NaH_2_PO_4_ (50 mm, pH 8.0), NaCl (300 mm), Triton X-100 (1%), and protease inhibitors (5 μg/μl) overnight at 4 °C. HisPur nickel-nitrilotriacetic acid spin columns (catalog no. 12393730, ThermoScientific) were prepared by washing with the binding buffer containing NaH_2_PO_4_ (50 mm, pH 8.0), NaCl (300 mm), Triton X-100 (1%), and glycerol (20%) before addition of the solubilized GABA_A_R subunit ECDs (∼800 μg) and incubation overnight at 4 °C. The following day, the columns were extensively washed to remove any unbound protein and Triton X-100 before elution of the bound proteins using elution buffer containing NaH_2_PO_4_ (50 mm, pH 6.0), NaCl (300 mm), protease inhibitors (5 μg/μl), glycerol (20%), EDTA (100 mm; catalog no. E9884, Sigma), and imidazole (500 mm; catalog no. 286874D, VWR) for 2 h (E1). A further two elutions (E2 and E3) were performed for 10 min, and all eluted fractions were dialyzed against PBS containing NaCl (300 mm) overnight at 4 °C. Protein concentration was measured, and proteins were further processed by bath sonication (ultra 7000 ultrasonic cleaner, Leighton Buzzard; Bedfordshire, UK) for 8 min followed by centrifugation at 21,190 × *g* for 10 min. Then, 5 μg of each of the prepared α1, α2, β2, and γ2 ECDs (0.36–0.4 μm) or the equivalent volume of Sf9 control extracts, which were processed using the same purification steps, were added to HEK293-MSN co-cultures within 30 min after plating. The cells were incubated for 24 h and fixed with 4% PFA.

##### Deglycosylation

To investigate whether the α1, α2, β2, and γ2 ECDs were produced as glycosylated proteins in Sf9 cells, a glycoprotein deglycosylation kit (catalog no. 362280, Millipore) was used. Briefly, 20 μg of each ECD was dissolved in 30 μl of deionized water in a microcentrifuge tube along with 10 μl of 5× reaction buffer (250 mm sodium phosphate buffer, pH 7.0) and 2.5 μl of denaturation solution (containing 2% SDS, 1 m β-mercaptoethanol, pH 7.0). Samples were boiled at 95 °C for 5 min and left to cool at room temperature. Then, 1 μl of the following enzymes endo-α-*N*-acetylgalactosaminidase, α2–3,6,8,9-neuraminidase, β1,4-galactosidase, β-*N*-acetylglucosaminidase, and *N*-glycosidase F were added to each of the samples, along with 2.5 μl of Triton X-100 detergent solution. Samples were incubated for 3 h at 37 °C before being analyzed using SDS-PAGE, alongside non-deglycosylated ECDs, as a positive control. Immunoblotting was carried out with a His_6_ tag primary antibody (1:1000; catalog no. AM1010a, Abgent) followed by the alkaline phosphatase-conjugated anti-rabbit secondary antibody (1:2500; catalog no. 31340, Invitrogen). Immunoreactivity was detected using the 5-bromo-4-chloro-3-indolyl phosphate toluidine salt (catalog no. S3771) and nitro blue tetrazolium chloride (catalog no. S3771) (both from Promega; Southampton, UK).

##### Electrophysiology

Whole-cell voltage clamp recordings of spontaneous inhibitory synaptic currents (sIPSCs) in α1β2-HEK293, α2β2-HEK293, α1β2γ2-HEK293, or α2β2γ2-HEK293 cells in co-culture with MSNs were made at a membrane potential of −60 mV (MultiClamp 700B with series resistance compensation; Molecular Devices). HEK293 cells expressing mCherry were identified based on their fluorescence using the X-Cite series 120Q light source (EXFO). The extracellular medium contained NaCl (130 mm), KCl (4 mm), HEPES (10 mm), NaHCO_3_ (20 mm), glucose (10 mm), MgCl_2_ (1 mm), and CaCl_2_ (2 mm) and was equilibrated with 5% CO_2_, 95% O_2_ (pH 7.4; 330 mosmol/liter; flow rate, 1.8 ml/min) at 32 °C. Patch electrodes had a final resistance of 3–8 megohms when filled with the intracellular solution containing KCl (130 mm), NaCl (3 mm), phosphocreatine (4.5 mm), HEPES (10 mm), EGTA (1 mm), Na-ATP (3.5 mm), Na-GTP (0.45 mm), and MgCl_2_ (2 mm) (adjusted to pH 7.2 with KOH, 290–300 mosmol/liter). GABA (125 μm; catalog no. 0344, Tocris Bioscience, Bristol, UK) was added to the bathing medium to test the response of the HEK293 cells expressing GABA_A_Rs as a control.

##### Immunoblotting

Protein samples were lysed with SDS (2%), and their concentration was determined using BCA assays. Samples were separated using SDS-PAGE and transferred onto a nitrocellulose membrane, which was then incubated with His_6_ tag antibody (1:1000; catalog no. AM1010a, Abgent) or the subunit-specific primary antibodies as follows: rabbit anti-GABA_A_R γ2 subunit (1:1000; catalog no. 224003, Synaptic Systems); anti-GABA_A_R β2/β3 subunit (1:50; UCL 112, raised against the N-terminal peptide of the β2 subunit in Dr. J. Jovanovic's laboratory); anti-GABA_A_R β3 subunit (1:500, UCL 74 (47)); anti-GABA_A_R α1 subunit (1:500 (46)); anti-GABA_A_R α2 subunit (1:500, catalog no. 224102, Synaptic Systems), or anti-GABA_A_R α2 subunit (2 μg/μl, raised against the intracellular loop (416–424) of the α2 subunit (48)). Membranes were washed and incubated with the alkaline phosphatase-conjugated anti-rabbit secondary antibody (1:2500; catalog no. 31340, Invitrogen) or the HRP-conjugated anti-rabbit antibody (1:1000; catalog no. 711-035-152-Jackson ImmunoResearch). Immunoreactivity was detected using 5-bromo-4-chloro-3-indolyl phosphate/nitro blue tetrazolium reagents or the SuperSignal West Femto Chemiluminescent substrate (catalog no. 34095, Fisher Scientific; Loughborough, UK), respectively.

## Results

### 

#### 

##### Formation of Functional GABAergic Synapses Is Initiated by the Expression of α1/β2/γ2-GABA_A_Rs at the Cell Surface

**FIGURE 1.**
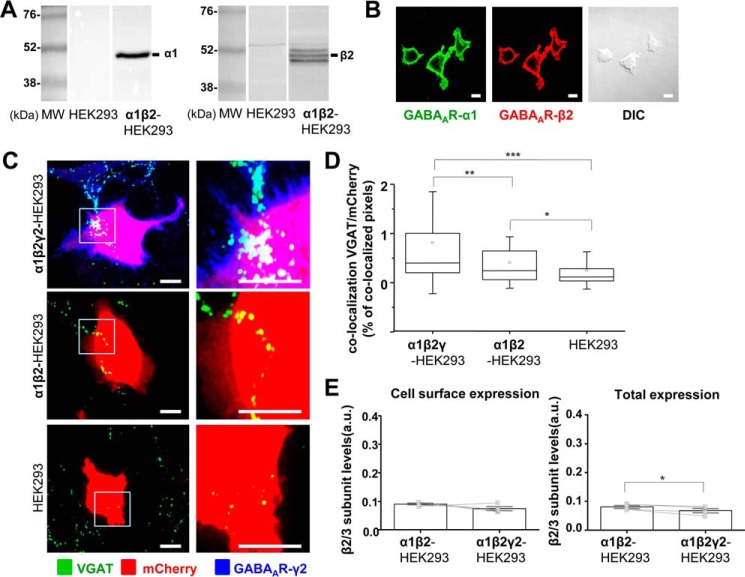
**Formation of synapse-like contacts between GABAergic MSNs and HEK293 cells expressing α1/β2/γ2-GABA_A_Rs at the cell surface.** *A,* immunoblot analysis of the expression of GABA_A_R α1 (*left panels*) and β2 (*right panels*) subunits in a stable α1β2-HEK293 cell line in comparison with the normal HEK293 cells, using specific primary antibodies and alkaline phosphatase-conjugated secondary antibodies for detection. Representative blots of *n* = 2 independent experiments are shown. *B,* immunocytochemical analysis of the expression of GABA_A_R α1 (*left panel*) and β2 (*middle panel*) subunits in a stable α1β2-HEK293 cell line, using primary antibodies specifically binding to the extracellular epitopes of the respective subunits and Alexa488-coupled (*left panel*) or Alexa555-coupled (*middle panel*) secondary antibodies. Differential interference contrast (*DIC*) image of the same field (*right panel*) is included for comparison. *Scale bar,* 10 μm. Representative images of *n* = 2 independent experiments are shown. *C,* immunolabeling of synaptic contacts between GABAergic presynaptic terminals of MSNs expressing VGAT and α1β2γ2-HEK293 cells (*top panel*), α1β2-HEK293 cells (*middle panel*), or HEK293 cells (*bottom panel*). GABAergic terminals were labeled with an anti-VGAT antibody (in *green*); HEK293 cells were labeled with mCherry (in *red*), and the surface-expressed GABA_A_Rs were labeled with the γ2 subunit-specific antibody (in *blue*). A selected area in each image (*left column, white box*) was magnified four times, and these images were included in the *right column. Scale bar,* 10 μm. *D,* quantification of contacts between MSNs and α1β2γ2-HEK293 cells, α1β2-HEK293 cells, or control HEK293 cells expressed as a percentage of co-localized pixels between VGAT-positive MSN terminals and mCherry-expressing HEK293 cells. The data were first analyzed using Shapiro-Wilk and Kolmogorov-Smirnov tests and subsequently using the non-parametric Mann-Whitney test with a confidence interval of 95%. The *box plots* display the median and interquartile range (*IQR*); *small squares* represent the mean, and *whiskers* represent the data range within 1 S.D. of the median (*n* = 62–77 cells from *n* = 6 independent experiments). *E,* cell surface levels of GABA_A_R β2 subunit in α1β2-HEK293 cells showed no significant change following transient transfection of the γ2 subunit (α1β2γ2-HEK293 cells), as measured using ELISA with an extracellular epitope-specific anti-β2/3 subunit antibody, followed by an HRP-conjugated secondary antibody and colorimetric reaction (*left panel*; *p* > 0.05, paired *t* test). Total levels of GABA_A_R β2 subunit in α1β2γ2-HEK293 cells were reduced (*right panel*; *p* < 0.05, paired *t* test). The *bar graphs* represent the mean ± S.D. of the total of *n* = 4 independent experiments. The superimposed paired scatterplots represent the individual data points obtained from α1β2-HEK293 and α1β2γ2-HEK293 cells. *, *p* < 0.05; **, *p* < 0.01; ***, *p* < 0.001.

To investigate the role of individual GABA_A_R subunits in synaptic contact formation, a new α1/β2-HEK293 stable cell line was generated and characterized by immunoblotting and immunocytochemistry using GABA_A_R subunit-specific antibodies (Fig. 1, *A* and *B*), revealing, respectively, the high level of expression of these subunits and their targeting to the cell surface. The α1/β2-HEK293 cells were further transiently transfected with mCherry reporter alone, to allow a reliable detection of these cells in co-culture, or in combination with the recombinant γ2 subunit cDNA (Fig. 1*C*, *top* and *middle panels*) and added to the primary cultures of MSN (14 DIV, Fig. 1*C*). Efficient assembly of the γ2 subunit with the α1 and β2 subunits, a prerequisite for the cell surface expression of this subunit (14, 15), was demonstrated by fluorescent labeling of this subunit at the cell surface with an antibody specific for its N-terminal extracellular epitope (Fig. 1*C*, *top panel*). In control experiments, HEK293 cells, which did not express any GABA_A_Rs, were transiently transfected with mCherry reporter and added to the MSN in culture (Fig. 1*C*, *bottom panel*). In all three conditions, cells were fixed after 24 h in co-culture and immunolabeled with a VGAT-specific antibody to identify the presynaptic MSN terminals, and a γ2 N-terminally specific antibody to detect the HEK293 cells expressing this subunit at the cell surface. Fluorescent labeling was analyzed using confocal microscopy (Fig. 1*C*), and co-localization between the presynaptic GABAergic, VGAT-positive terminals, and the HEK293 cells, labeled with mCherry, was quantified using ImageJ (Fig. 1*D*). Multiple synapse-like contacts were represented by presynaptic VGAT-positive terminals residing on the surface of α1β2- or α1β2γ2-HEK293 cells (Fig. 1*C*, *middle* and *top panel*) and therefore co-localizing with the mCherry, as previously extensively characterized (33). In contrast, only a few sporadic terminals were found on the surface of control HEK293 cells (Fig. 1*C*, *bottom panel*). Quantification with ImageJ revealed that the degree of innervation of HEK293 cells, represented by the percentage of co-localized pixels between VGAT-positive MSN terminals and mCherry expressing HEK293 cells, was significantly higher when γ2 subunit was incorporated into the GABA_A_Rs (0.40% IQR = 0.20–1.05; *n* = 77 cells, *versus* 0.24% IQR = 0.06–0.66; *n* = 62 cells; *p* < 0.01, Mann-Whitney test; *n* = 6 independent experiments). However, α1β2-GABA_A_R were also able to induce synaptic contact formation, albeit at much lower level, yielding a significant increase in the percentage of co-localized pixels between VGAT terminals and mCherry-expressing HEK293 cells (0.24% IQR = 0.06–0.66; *n* = 62 cells, *versus* 0.12% IQR = 0.03–0.28; *n* = 62 cells; *p* < 0.05; *n* = 6 independent experiments).

To assess whether the effects of the γ2 subunit in synaptic contact formation were observed because the presence of this subunit increased the stability and the overall cell surface expression of GABA_A_Rs, cell surface ELISA experiments were performed. In these experiments, cell surface and total expression levels of the β2 GABA_A_R subunit were monitored using a β2/3-subunit-specific antibody, which binds to an epitope in the N-terminal extracellular domain of either of these subunits. This approach was chosen because the incorporation of the β subunits is required for the appropriate assembly of GABA_A_Rs and their forward trafficking to the plasma membrane (16). Their detection at the cell surface is therefore a reliable indicator of the overall surface expression of GABA_A_Rs. These experiments demonstrated that the presence of the γ2 subunit did not affect the cell surface expression of GABA_A_Rs (*p* > 0.05, paired *t* test), although the total expression of GABA_A_Rs was slightly reduced (*p* < 0.05, paired *t* test; Fig. 1*E*). In addition, immunolabeling using the same β2/3 subunit-specific antibody in combination with the α1-specific antibody demonstrated that the pattern of cell surface expression of GABA_A_Rs in the presence of γ2 subunit remained unaltered, showing uniform distribution of these receptors in both conditions (data not shown). These results have indicated that γ2 subunit plays an important role in the efficient formation of synaptic contacts triggered by GABA_A_Rs.

**FIGURE 2.**
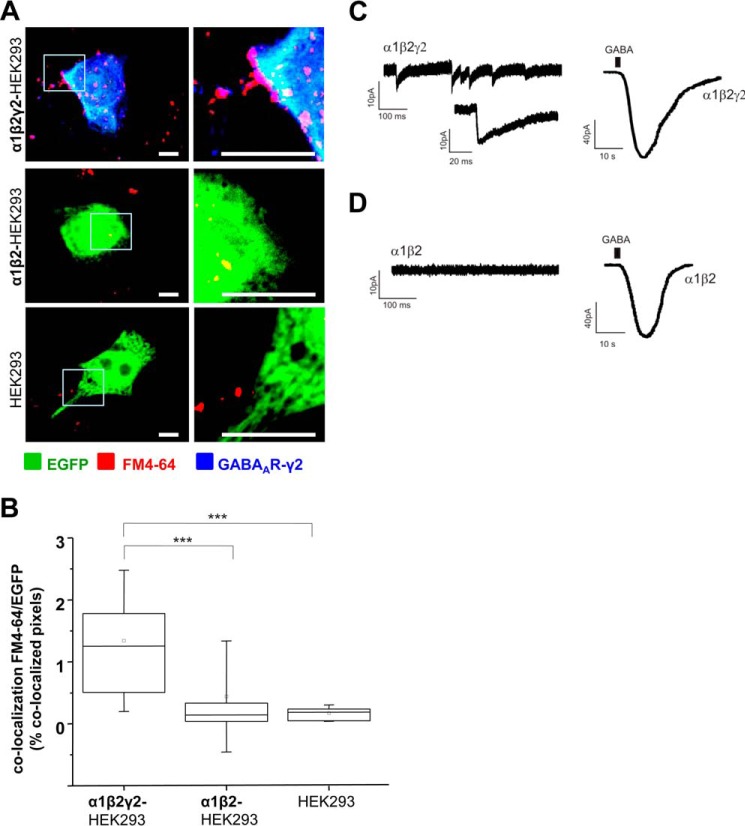
**Synapse-like contacts formed between GABAergic MSNs and HEK293 cells expressing α1/β2/γ2-GABA_A_Rs are active and capable of supporting spontaneous postsynaptic GABAergic currents.** *A,* activity-dependent uptake of FM4-64FX (*red*) into presynaptic MSN terminals forming contacts with α1β2γ2-HEK293 cells (*top panel*), α1β2-HEK293 cells (*middle panel*), or HEK293 cells (*bottom panel*). HEK293 cells were labeled with EGFP (in *green*), and the surface-expressed GABA_A_Rs were labeled with the γ2 subunit-specific antibody (in *blue*). A selected area in each image (*left column, white box*) was magnified four times, and these images were included in the *right column. Scale bar,* 10 μm. *B,* quantification of active synaptic contacts between MSN terminals and α1β2γ2-HEK293 cells, α1β2-HEK293 cells, or control HEK293 cells expressed as a percentage of co-localized pixels between FM4-64FX and EGFP-expressing HEK293 cells. The data were first analyzed using Shapiro-Wilk and Kolmogorov-Smirnov tests and subsequently using the non-parametric Mann-Whitney test with the confidence interval of 95%. The *box plots* display the median and IQR; *small squares* represent the mean, and *whiskers* represent the data range within 1 S.D. of the median (*n* = 16–17 cells from *n* = 2 independent experiments). *C,* recordings of sIPSCs in an α1β2γ2-HEK293 cell innervated by MSN axons in control medium (*left trace*), followed by the whole-cell response to exogenously applied GABA to the bath (1 mm, *right trace*). Example traces from *n* = 2 independent experiments. *D,* recordings from a α1β2-HEK293 cell co-cultured with MSNs in control medium (*left trace*), followed by the whole-cell response to exogenously applied GABA to the bath (1 mm, *right trace*). Example traces from *n* = 2 independent experiments. ***, *p* < 0.001.

That these synaptic contacts are active in releasing GABA was demonstrated by efficient activity-dependent incorporation of the lipophilic dye, FM4-64FX, into the small synaptic vesicles undergoing neurotransmitter release from the presynaptic terminals (Fig. 2*A*). In the presence of α1β2γ2-GABA_A_Rs, incorporation of FM4-64 into terminals forming synapses with HEK293 cells was very prominent (Fig. 2*A*, *top panel*) and showed a significant increase in comparison with α1β2-GABA_A_R-expressing HEK293 cells (Fig. 2*A*, *middle panel*) or control HEK293 cells (Fig. 2*A*, *bottom panel*). Quantification with ImageJ was consistent with the observed increase in labeling of active terminals. The percentage of co-localized pixels between FM4-64FX-positive terminals and EGFP-expressing α1β2γ2-HEK293 cells (1.26% IQR = 0.45–2.00; *n* = 17) was significantly higher than in the case of α1β2-HEK293 cells (0.14% IQR = 0.04–0.37; *n* = 16 cells, *p* < 0.001) or control HEK293 cells (0.19% IQR = 0.04–0.24; *n* = 17 cells, *p* < 0.001, from *n* = 2 independent experiments) (Fig. 2*B*).

Whole-cell recordings from α1β2γ2-HEK293 cells in co-culture revealed that these cells formed functional synaptic contacts with MSNs that were able to support spontaneous postsynaptic GABAergic currents with the frequency of 8.7 ± 3.6 Hz (*n* = 4 from *n* = 2 independent experiments; Fig. 2*C*, *left panel* and *inset*). In contrast, recordings from α1β2-HEK293 cells co-cultured with MSNs showed no synaptic activity (Fig. 2*D*, *left panel*). In both cases, HEK293 cells were able to respond robustly to application of the exogenous GABA, further confirming the efficient cell surface expression of GABA_A_Rs (Fig. 2, *C* and *D*, *right panel*).

##### Formation of Functional GABAergic Synapses Is Also Initiated by the Cell Surface Expression of α2/β2/γ2-GABA_A_Rs

**FIGURE 3.**
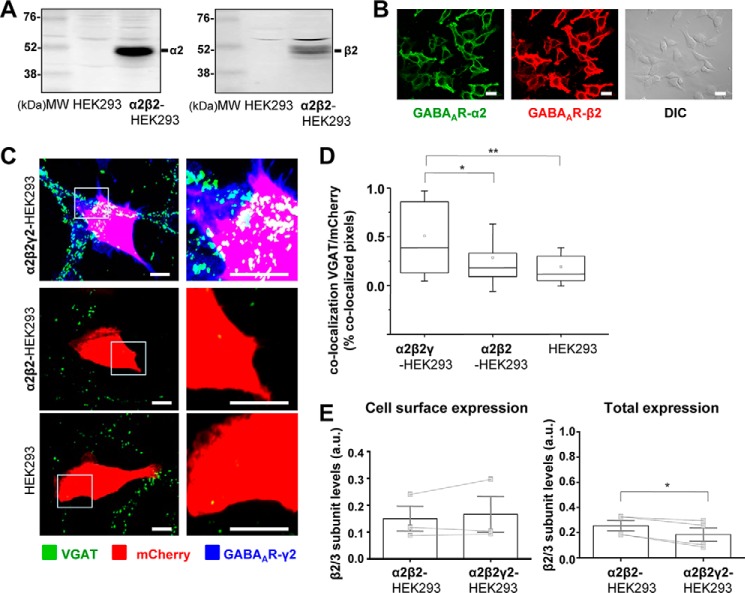
**Formation of synapse-like contacts between GABAergic MSNs and HEK293 cells expressing α2/β2/γ2-GABA_A_Rs at the cell surface.** *A,* immunoblot analysis of the expression of GABA_A_R α2 (*left panel*) and β2 (*right panel*) subunits in a stable α2β2-HEK293 cell line in comparison with the normal HEK293 cells, using specific primary antibodies and alkaline phosphatase-conjugated secondary antibodies for detection. Representative blots of *n* = 2 independent experiments are shown. *B,* immunocytochemical analysis of the expression of GABA_A_R α2 (*left panel*) and β2 (*middle panel*) subunits in a stable α2β2-HEK293 cell line, using primary antibodies specifically binding to the extracellular epitopes of the respective subunits and Alexa488-coupled (*left panel*) or Alexa555-coupled (*middle panel*) secondary antibodies. DIC image of the same field (*right panel*) is included for comparison. *Scale bar,* 10 μm. Representative images of *n* = 2 independent experiments are shown. *C,* immunolabeling of synaptic contacts between GABAergic presynaptic terminals of MSNs expressing VGAT and α2β2γ2-HEK293 cells (*top panel*), α2β2-HEK293 cells (*middle panel*), or HEK293 cells (*bottom panel*). GABAergic terminals were labeled with an anti-VGAT antibody (in *green*); HEK293 cells were labeled with mCherry (in *red*), and the surface-expressed GABA_A_Rs were labeled with the γ2 subunit-specific antibody (in *blue*). A selected area in each image (*left column, white box*) was magnified four times, and these images are included in the *right column. Scale bar,* 10 μm. *D,* quantification of contacts between MSNs and α2β2γ2-HEK293 cells, α2β2-HEK293 cells or control HEK293 cells expressed as a percentage of co-localized pixels between VGAT positive MSN terminals and mCherry expressing HEK293 cells. The data were first analyzed using Shapiro-Wilk and Kolmogorov-Smirnov tests and subsequently using the non-parametric Mann-Whitney test with a confidence interval of 95%. The *box plots* display the median and IQR; *small squares* represent the mean, and *whiskers* represent the data range within 1 S.D. of the median (*n* = 42–56 cells from *n* = 3 independent experiments). *E,* cell surface levels of GABA_A_R β2 subunit in α2β2-HEK293 cells showed no significant change following transient transfection of the γ2 subunit (α2β2γ2-HEK293 cells), as measured using ELISA with an extracellular epitope-specific anti-β2/3 subunit antibody, followed by an HRP-conjugated secondary antibody and colorimetric reaction (*left panel*; *p* > 0.05, paired *t* test). Total levels of GABA_A_R β2 subunit in α2β2γ2-HEK293 cells were reduced (*right panel*; *p* < 0.05, paired *t* test). The *bar graphs* represent the mean ± S.D. of the total of *n* = 3 (for cell surface expression) and *n* = 4 (for total expression) independent experiments. The superimposed paired scatterplots represent the individual data points obtained from α2β2-HEK293 and α2β2γ2-HEK293 cells. *, *p* < 0.05; **, *p* < 0.01.

To gain further insight into the molecular mechanisms mediating the initiation of GABAergic synapses, a stable cell line expressing the α2β2-GABA_A_Rs was generated and characterized using immunoblotting and immunocytochemistry (Fig. 3, *A* and *B*) revealing, respectively, the high level of expression of both receptor subunits and their targeting to the cell surface.

To assess the role of individual GABA_A_R subunits in synaptic contact formation, the α2β2-HEK293 cells were transiently transfected with mCherry and γ2 subunit constructs or with mCherry construct alone, plated into the primary cultures of MSNs at 14 DIV, and incubated for 24 h. Cells were subsequently fixed, and surface GABA_A_Rs were labeled with an extracellular epitope-specific γ2 antibody, while the presynaptic MSN terminals were immunolabeled for VGAT, as described above. Synaptic contacts were identified based on co-localization between the presynaptic VGAT-positive terminals and the mCherry-expressing HEK293. Similar to the results obtained with the α1β2γ2-HEK293 cells, the presynaptic VGAT terminals were observed as multiple puncta residing on the surface of α2β2γ2-HEK293 cells (Fig. 3*C*, *top panel*). However, in contrast to the previous results with α1β2-HEK293 cells, synaptic contacts between VGAT terminals and α2β2-HEK293 cells (Fig. 3*C*, *middle panel*) did not form. ImageJ analysis revealed that inclusion of the γ2 subunit significantly increased the percentage of co-localized pixels between VGAT terminals and mCherry expressing α2β2γ2-HEK293 cells compared with the control HEK293 cells (0.39% IQR = 0.12–0.87; *n* = 28 cells *versus* 0.12% IQR = 0.05–0.30; *n* = 56 cells; *p* < 0.01), or compared with the α2β2-HEK293 cells (0.18% IQR = 0.08–0.34; *p* < 0.05; *n* = 42 cells; all from *n* = 2 to 3 independent experiments).

To test whether the positive effects of the γ2 subunit on synaptic contact formation were observed because of the increased cell surface expression of GABA_A_Rs, cell surface ELISA experiments were performed with the same extracellular epitope-specific β2/3 subunit antibody as described above. These experiments revealed that cell surface expression of GABA_A_Rs did not change significantly in the presence of the γ2 subunit, although total expression of GABA_A_Rs showed a small but statistically significant reduction (*p* < 0.05, paired *t* test; Fig. 3*E*). These data thus indicate that induction of synaptic contacts was caused by incorporation of the γ2 subunit into the receptor. Distribution of GABA_A_Rs at the cell surface was also unchanged following the incorporation of the γ2 subunit, as revealed by immunolabeling with β2/3- and α2-specific antibodies that bound the extracellular N-terminal domains of these subunits (data not shown).

**FIGURE 4.**
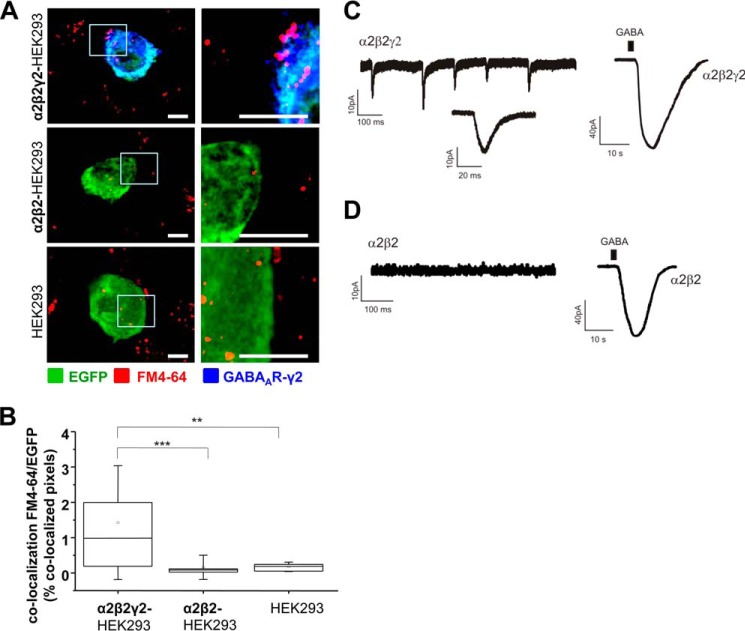
**Synapse-like contacts formed between GABAergic MSNs and HEK293 cells expressing α2/β2/γ2-GABA_A_Rs are active and capable of supporting spontaneous postsynaptic GABAergic currents.** *A,* activity-dependent uptake of FM4-64FX (*red*) into presynaptic MSN terminals forming contacts with α2β2γ2-HEK293 cells (*top panel*), α2β2-HEK293 cells (*middle panel*), or HEK293 cells (*bottom panel*). HEK293 cells were labeled with EGFP (in *green*), and the surface expressed GABA_A_Rs were labeled with the γ2 subunit-specific antibody (in *blue*). A selected area in each image (*left column, white box*) was magnified four times, and these images were included in the *right column. Scale bar,* 10 μm. *B,* quantification of active synaptic contacts between MSN terminals and α1β2γ2-HEK293 cells, α1β2-HEK293 cells, or control HEK293 cells expressed as a percentage of co-localized pixels between FM4-64FX and EGFP-expressing HEK293 cells. The data were first analyzed using Shapiro-Wilk and Kolmogorov-Smirnov tests and subsequently using the non-parametric Mann-Whitney test with the confidence interval of 95%. The *box plots* display the median and IQR; *small squares* represent the mean, and *whiskers* represent the data range within 1 S.D. of the median (*n* = 16–17 cells from *n* = 2 independent experiments). *C,* recordings of sIPSCs in a α2β2γ2-HEK293 cell innervated by MSN axons in control medium (*left trace*), followed by the whole-cell response to exogenously applied GABA to the bath (1 mm, *right trace*). Example traces from *n* = 3 independent experiments. *D,* recordings from a α2β2-HEK293 cell co-cultured with MSNs in control medium (*left trace*), followed by the whole-cell response to exogenously applied GABA to the bath (1 mm, *right trace*). Example traces from *n* = 2 independent experiments. **, *p* < 0.01; ***, *p* < 0.001.

The majority of synaptic contacts formed between MSNs and α2β2γ2-HEK293 cells were functional as they showed the activity-dependent incorporation of FM4-64FX dye into the presynaptic GABA-releasing terminals (Fig. 4*A*), which appeared as fluorescent puncta on the surface of EGFP-labeled HEK293 cell. Quantification using ImageJ showed a significant increase in the percentage of co-localized pixels between FM4-64FX-positive MSN terminals and EGFP-expressing α2β2γ2-HEK293 cells (0.99% IQR = 0.19–2.05; *n* = 16 cells) in comparison with α2β2-HEK293 cells (0.08% IQR = 0.02–0.12; *n* = 16; *p* < 0.001) or control HEK293 cells (0.19% IQR = 0.04–0.24; *n* = 17 cells; *p* < 0.01; all from *n* = 2 independent experiments; Fig. 4*B*). Whole-cell recordings from α2β2γ2-HEK293 cells (Fig. 4*C*, *left panel* and *inset*) confirmed that these contacts were active as they were able to support the spontaneous postsynaptic GABAergic currents (IPSCs) with the frequency of 5 ± 1 Hz (*n* = 3 from *n* = 3 independent experiments). IPSCs were not detected in recordings from α2β2-HEK293 cells (Fig. 4*D*, *left panel*), indicating that these cells were not innervated by MSN axons. However, both α2β2γ2-HEK293 and α2β2-HEK293 cells responded robustly to application of the exogenous GABA (Fig. 4, *C* and *D*, *right panel*) in control experiments, demonstrating that GABA_A_Rs were expressed at the cell surface.

Together, these experiments reveal that incorporation of the γ2 subunit into the GABA_A_R is critical for the induction of active synaptic contacts in this system. However, these results also suggest that the type of the α subunit co-assembled with the β2 and γ2 subunits influences this process as the α1-containing combination appeared to be more potent in inducing synaptic contacts than the α2 combination (6.9-fold *versus* 5.4-fold increase, both normalized with the respective control HEK293 cells).

##### Type of the β Subunit Present in the GABA_A_R Influences the Formation of Synaptic Contacts

**FIGURE 5.**
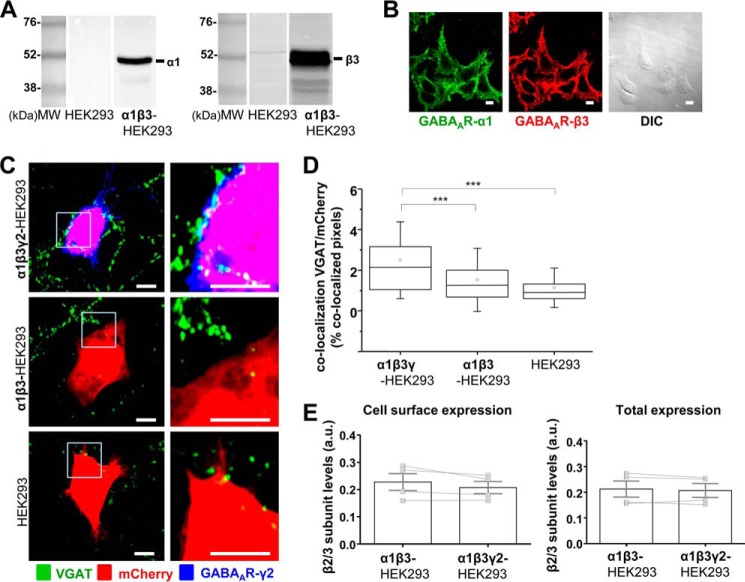
**Formation of synapse-like contacts between GABAergic MSNs and HEK293 cells expressing α1/β3/γ2-GABA_A_Rs at the cell surface.** *A,* immunoblot analysis of the expression of GABA_A_R α1 (*left panel*) and β3 (*right panel*) subunits in a stable α1β3-HEK293 cell line in comparison with the normal HEK293 cells, using specific primary antibodies and alkaline phosphatase-conjugated secondary antibodies for detection. Representative blots of *n* = 2 independent experiments are shown. *B,* immunocytochemical analysis of the expression of GABA_A_R α1 (*left panel*) and β3 (*middle panel*) subunits in a stable α1β3-HEK293 cell line, using primary antibodies specifically binding to the extracellular epitopes of the respective subunits and Alexa488-coupled (*left panel*) or Alexa555-coupled (*middle panel*) secondary antibodies. DIC image of the same field (*right panel*) is included for comparison. *Scale bar,* 10 μm. Representative images of *n* = 2 independent experiments are shown. *C,* immunolabeling of synaptic contacts between GABAergic presynaptic terminals of MSNs expressing VGAT and α1β3γ2-HEK293 cells (*top panel*), α1β3-HEK293 cells (*middle panel*), or HEK293 cells (*bottom panel*). GABAergic terminals were labeled with an anti-VGAT antibody (in *green*); HEK293 cells were labeled with mCherry (in *red*); and the surface-expressed GABA_A_Rs were labeled with the γ2 subunit-specific antibody (in *blue*). A selected area in each image (*left column, white box*) was magnified four times, and these images were included in the *right column. Scale bar,* 10 μm. *D,* quantification of contacts between MSNs and α1β3γ2-HEK293 cells, α1β3-HEK293 cells or control HEK293 cells expressed as a percentage of co-localized pixels between VGAT-positive MSN terminals and mCherry-expressing HEK293 cells. The data were first analyzed using Shapiro-Wilk and Kolmogorov-Smirnov tests and subsequently using the non-parametric Mann-Whitney test with a confidence interval of 95%. The *box plots* display the median and IQR; *small squares* represent the mean, and *whiskers* represent the data range within 1 S.D. of the median (*n* = 37–49 cells from *n* = 4 independent experiments). *E,* cell surface levels of GABA_A_R β3 subunit in α1β3-HEK293 cells showed no significant change following transient transfection of the γ2 subunit (α1β3γ2-HEK293 cells), as measured using ELISA with an extracellular epitope-specific anti-β2/3 subunit antibody, followed by an HRP-conjugated secondary antibody and colorimetric reaction (*left panel*; *p* > 0.05, paired *t* test). The *bar graphs* represent the mean ± S.D. of the total of *n* = 4 independent experiments. The superimposed paired scatterplots represent the individual data points obtained from α1β3-HEK293 and α1β3γ2-HEK293 cells. ***, *p* < 0.001.

As both the α1β2γ2-GABA_A_R and α2β2γ2-GABA_A_R were able to promote synapse formation, the effects of the β3-containing GABA_A_Rs expressed stably in HEK293 cells were next investigated. A new stable cell line expressing the α1β3-GABA_A_Rs was generated and characterized using immunoblotting and immunocytochemistry as described above. A high level of expression of the α1 and β3 subunits was detected using specific antibodies (Fig. 5*A*). Immunolabeling with the same antibodies indicated that both subunits were expressed at the cell surface in all the cells examined (Fig. 5*B*).

The α1β3-HEK293 cells were transiently transfected with mCherry and γ2 subunit construct or mCherry construct alone, plated into the primary cultures of MSNs at 14 DIV, and incubated for 24 h. Cells in co-culture were fixed, and surface GABA_A_Rs were labeled with the extracellular epitope-specific γ2 antibody, as described above, and the presynaptic MSN terminals were immunolabeled for VGAT. Synaptic contacts were identified based on co-localization between the presynaptic VGAT-positive terminals and the mCherry-expressing HEK293 (Fig. 5*C*) and quantified using ImageJ (Fig. 5*D*). Similar to the previous results, incorporation of the γ2 subunit was a pre-requisite for synaptic contact formation (co-localization = 2.14% IQR = 1.05–3.18; *n* = 48 cells; Fig. 5, *C* and *D*), as only very rare, sporadic contacts were observed between the presynaptic terminals labeled with VGAT and the α1β3-HEK293 cells (co-localization = 1.26% IQR = 0.66–2.05; *n* = 49 cells) or control HEK293 cells (co-localization = 0.90% IQR = 0.58–1.32; *n* = 37 cells; Fig. 5, *C* and *D*). The difference in the percentage of co-localization between VGAT terminals and α1β3γ2-HEK293 cells *versus* α1β3-HEK293 cells or control HEK293 cells was statistically significant (*p* < 0.001; from *n* = 4 independent experiments).

In agreement with the previous experiments (Figs. 1*E* and 3*E*), the overall cell surface and total expression levels of GABA_A_Rs in α1β3-HEK293 cells were not significantly altered following the incorporation of the γ2 subunit (Fig. 5*E*), as demonstrated using cell surface ELISA with the same anti-β2/3 subunit antibody as described above. Likewise, distribution of these receptors at the cell surface was also unaltered as revealed by immunolabeling with the specific antibodies as described above (data not shown).

**FIGURE 6.**
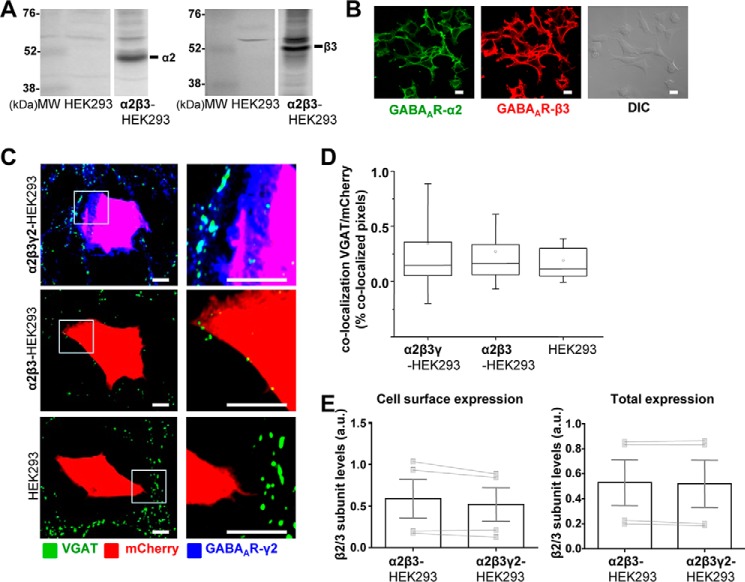
**Synapse-like contacts do not form between GABAergic MSNs and HEK293 cells expressing α2/β3/γ2-GABA_A_Rs at the cell surface.** *A,* immunoblot analysis of the expression of GABA_A_R α2 (*left panel*) and β3 (*right panel*) subunits in a stable α2β3-HEK293 cell line in comparison with the normal HEK293 cells, using specific primary antibodies and alkaline phosphatase-conjugated secondary antibodies for detection. Representative blots of *n* = 2 independent experiments are shown. *B,* immunocytochemical analysis of the expression of GABA_A_R α2 (*left panel*) and β3 (*middle panel*) subunits in a stable α2β3-HEK293 cell line, using primary antibodies specifically binding to the extracellular epitopes of the respective subunits and Alexa488- (*left panel* or Alexa555-coupled (*middle panel*)) secondary antibodies. DIC image of the same field (*right panel*) is included for comparison. *Scale bar*, 10 μm. Representative images of *n* = 2 independent experiments are shown. *C,* immunolabeling of synaptic contacts between GABAergic presynaptic terminals of MSNs expressing VGAT and α2β3γ2-HEK293 cells (*top panel*), α2β3-HEK293 cells (*middle panel*), or HEK293 cells (*bottom panel*). GABAergic terminals were labeled with an anti-VGAT antibody (in *green*); HEK293 cells were labeled with mCherry (in *red*), and the surface expressed GABA_A_Rs were labeled with the γ2 subunit-specific antibody (in *blue*). A selected area in each image (*left column, white box*) was magnified four times, and these images were included in the *right column. Scale bar,* 10 μm. *D,* quantification of contacts between MSNs and α2β3γ2-HEK293 cells, α2β3-HEK293 cells, or control HEK293 cells expressed as a percentage of co-localized pixels between VGAT-positive MSN terminals and mCherry-expressing HEK293 cells. The data were first analyzed using Shapiro-Wilk and Kolmogorov-Smirnov tests and subsequently using the non-parametric Mann-Whitney test with a confidence interval of 95%. The *box plots* display the median and IQR; *small squares* represent the mean, and *whiskers* represent the data range within 1 S.D. of the median (*n* = 42–56 cells from *n* = 3 independent experiments). *E,* cell surface levels of GABA_A_R β3 subunit in α2β3-HEK293 cells showed no significant change following transient transfection of the γ2 subunit (α2β3γ2-HEK293 cells), as measured using ELISA with an extracellular epitope-specific anti-β2/3 subunit antibody, followed by an HRP-conjugated secondary antibody and colorimetric reaction (*left panel*; *p* > 0.05, paired *t* test). The *bar graphs* represent the mean ± S.D. of the total of *n* = 4 independent experiments. The superimposed paired scatterplots represent the individual data points obtained from α2β3-HEK293 and α2β3γ2-HEK293 cells.

To further test the effects of different β subunits in synaptic contact formation, a new α2β3-HEK293 stable cell line was generated, and the expression of each GABA_A_R subunit was characterized by immunoblotting with the specific antibodies (Fig. 6*A*). Expression of both subunits at the cell surface was demonstrated using immunocytochemistry with the antibodies specific for their extracellular N-terminal domains (Fig. 6*B*).

Following the procedure described above, the α2β3-HEK293 cells were transiently transfected with mCherry and γ2 subunit constructs or with mCherry construct alone, plated into the primary cultures of MSNs at 14 DIV, and incubated for 24 h. Cells in co-culture were fixed and labeled with an extracellular epitope-specific γ2 antibody, whereas the presynaptic MSN terminals were immunolabeled for VGAT. In contrast to all other combinations of α, β, and γ2 subunits tested so far, the α2β3γ2 combination expressed in HEK293 cells failed to promote synaptic contact formation with co-cultured MSNs. Based on co-localization between the presynaptic VGAT terminals and mCherry-expressing HEK293 cells, very rare or no synaptic contacts were observed in co-cultures containing either α2β3γ2-, α2β3-, or control HEK293 cells (Fig. 6*C*). Quantification using ImageJ was consistent with this observation, revealing no statistically significant difference in the percentage of co-localized pixels between VGAT terminals and α2β3γ2-, α2β3-, or control mCherry-HEK293 cells (0.15% IQR = 0.05–0.36; *n* = 42 cells, *versus* 0.16% IQR = 0.06–0.34; *n* = 42 cells, *versus* 0.12% IQR = 0.05–0.30; *n* = 56 cells; *p* > 0.05 in all comparisons; from *n* = 3 independent experiments).

As with all other combinations of the receptor subunits tested using cell surface ELISA, incorporation of the γ2 subunit did not significantly change the overall cell surface or total expression of GABA_A_Rs (Fig. 6*E*) or their immunolabeling at the cell surface (data not shown). Together, the data indicate that although the γ2 subunit is required for the efficient initiation of synaptic contacts in this system, the co-assembled α and β subunits have a permissive role in this process, not only because they are necessary for the forward trafficking of the γ2 subunit to the cell surface, but also because they appear to influence the extent of contact formation triggered in the presence of the γ2 subunit.

##### Synaptic Contact Formation Was Not Dependent on the Activity of GABA_A_Rs

**FIGURE 7.**
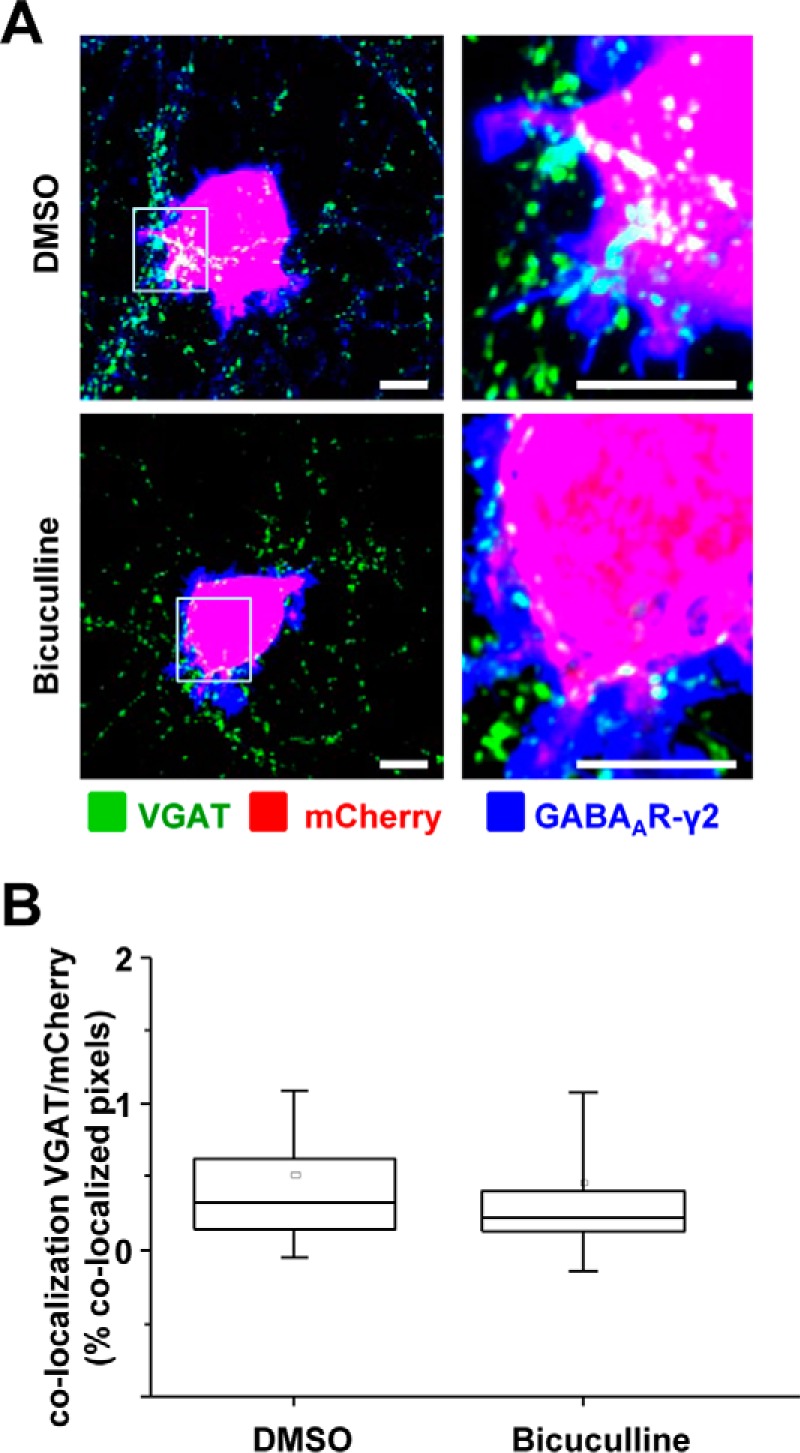
**Inhibition of GABA_A_R activity by application of bicuculline has no effect on the formation of synapse-like contacts between MSNs and α1β2γ2-HEK293 cells.** *A,* immunolabeling of synaptic contacts between the presynaptic terminals of MSNs and α1β2γ2-HEK293 cells in co-cultures treated with DMSO (*upper panel*) or bicuculline (*lower panel*). GABAergic terminals were labeled with an anti-VGAT antibody (in *green*); HEK293 cells were labeled with mCherry (in *red*), and the surface-expressed GABA_A_Rs were labeled with the γ2 subunit-specific antibody (in *blue*). A selected area in each image (*left column, white box*) was magnified four times, and these images were included in the *right column. Scale bar,* 10 μm. *B,* quantification of contacts between MSNs and α1β2γ2-HEK293 cells expressed as percentage of co-localized pixels between VGAT-positive terminals and mCherry expressing HEK293 cells. The Gaussian distribution of the data was initially tested using Shapiro-Wilk and Kolmogorov-Smirnov tests. The data were subsequently analyzed using the non-parametric Mann-Whitney test with a confidence interval of 95%. The *box plots* display the median and IQR; *small squares* represent the mean, and *whiskers* represent the data range within 1 S.D. of the median of (*n* = 38–40 cells from three independent experiments).

To test whether the GABA_A_R-triggered formation of synaptic contacts was dependent on activation of these receptors by GABA, a specific antagonist bicuculline (25 μm) was applied immediately following the plating of the α1β2γ2-HEK293 cells into the MSN cultures. The cells were fixed after 24 h and immunolabeled as described above. Synaptic contact formation indicated by the multiple puncta of VGAT terminals residing on the surface of mCherry-labeled HEK293 cells was observed in both the vehicle (DMSO)- and bicuculline-treated co-cultures (Fig. 7*A*). In ImageJ analysis, the percentage of co-localized pixels between the VGAT terminals and mCherry-HEK293 cells was not significantly different between DMSO- and bicuculline-treated co-cultures (0.32% IQR = 0.14–0.62; *n* = 40, *versus* 0.22% IQR = 0.12–0.40; *n* = 37, from *n* = 3 independent experiments; *p* > 0.05; Fig. 7*B*). These experiments indicate that the ability of GABA_A_Rs to initiate synaptic contacts is independent from their activation by GABA and chloride current gating and suggest that these receptors play a structural role similar to the previously described role of synaptic adhesion proteins.

##### N-terminal Extracellular Domains of GABA_A_Rs Directly Participate in Synaptic Contact Formation

To further investigate the mechanisms underlying the initiation of synaptic contacts by GABA_A_Rs, we cloned, expressed, and purified the N-terminal extracellular domains of α1, α2, β2, and γ2 subunits using the baculovirus/Sf9 cell expression system. The N-terminal extracellular domains of GABA_A_R subunits are very large and complex and also are conserved in their primary amino acid sequence between and within different subunit classes (4).

**FIGURE 8.**
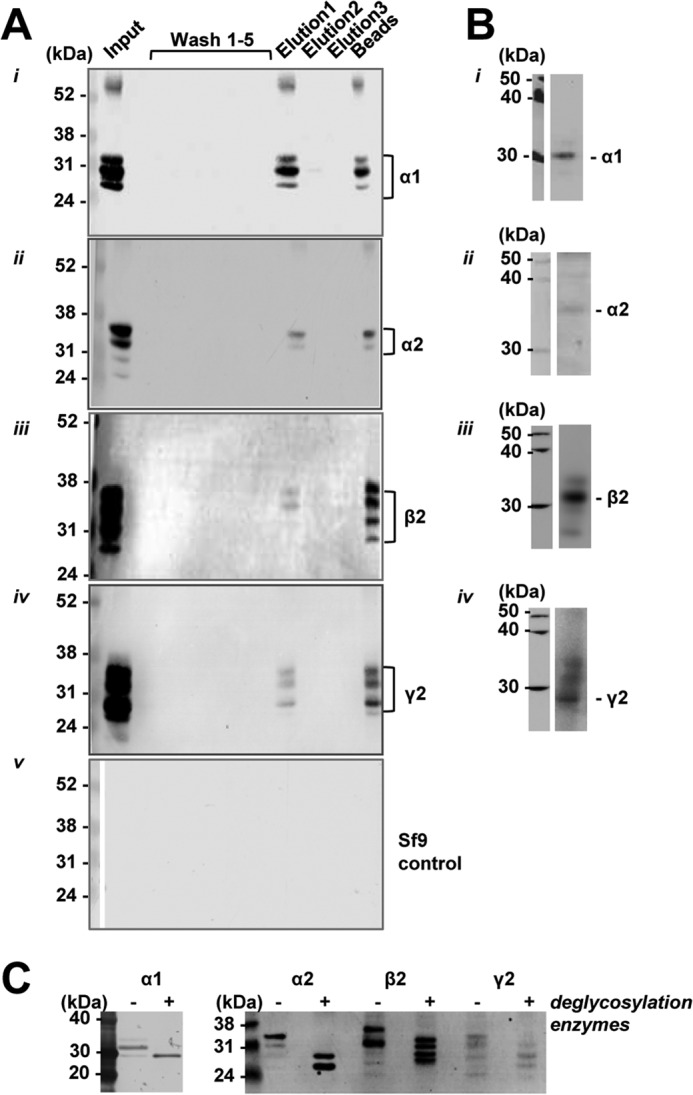
**Purification of N-terminal ECDs of GABA_A_R subunits using affinity chromatography.** *A,* samples were collected throughout the purification of N-terminal ECDs of GABA_A_R α1 (*panel i*), α2 (*panel ii*), β2 (*panel iii*), and γ2 subunit (*panel iv*) from baculovirus-infected Sf9 cells extracts or control Sf9 cell extracts (*panel v*) and analyzed by SDS-PAGE and immunoblotting using an anti-His_6_ tag antibody and alkaline phosphatase-conjugated secondary antibodies. From *left* to *right*, *input lane*, *washes 1–5*, *elutions 1–3,* and *beads* after elution. *B,* purified N-terminal ECDs (5 ug) of α1 (*panel i*), α2 (*panel ii*), β2 (*panel iii*), or γ2 subunit (*panel iv*) were analyzed by immunoblotting using subunit-specific antibodies and HRP-conjugated secondary antibodies. *C,* purified N-terminal ECDs of α1, α2, β2, and γ2 subunits were incubated with deglycosylation enzymes and analyzed by immunoblotting with anti-His_6_ tag antibody and alkaline phosphatase-conjugated secondary antibodies.

Purification of N-terminal ECDs from the baculovirus-infected Sf9 cells was carried out under sterile conditions as described under “Experimental Procedures.” Solubilization of proteins from Sf9 cells (Fig. 8*A*, *panels i–v, input*) allowed extraction of a significant amount of all N-terminal ECDs. These proteins were subsequently dialyzed and bound to the prepared nickel-nitrilotriacetic acid columns for purification by affinity chromatography. The elution of the bound ECDs was carried out using an imidazole/EDTA-containing buffer yielding a relatively small amount of purified proteins (∼200 μg) but sufficient to carry out further experiments (Fig. 8*A, panels i–v, elution*). Small samples from each purification step were collected and analyzed by SDS-PAGE and immunoblotting using subunit ECD-specific antibodies. The immunoreactivity detected in blots (Fig. 8*B*, *panels i–iv*) confirmed that these proteins were indeed correctly expressed in Sf9 cells and that their detected molecular mass (∼30–36 kDa) was compatible with that predicted on the basis of their amino acid sequence. However, the presence of multiple bands running closely in SDS-PAGE, suggested that they may represent glycosylated forms of the N-terminal ECDs, given that glycosylation of exogenous proteins in Sf9 cells has been demonstrated (49–52) and that the number of predicted glycosylation sites for the N-terminal ECDs is compatible with the number of detected protein bands (two residues predicted in the α1 subunit and three in the β2 and γ2 subunit N-terminal ECDs). This was confirmed by incubating purified ECDs with deglycosylation enzymes and analyzing them by SDS-PAGE and immunoblotting with His tag antibodies. All four ECDs showed a change in the pattern of immunoreactive bands and a reduction in their molecular mass due to removal of glycosyl groups (Fig. 8*C*).

##### α1, β2, and γ2 ECD Each Contribute to Synaptic Contact Formation

**FIGURE 9.**
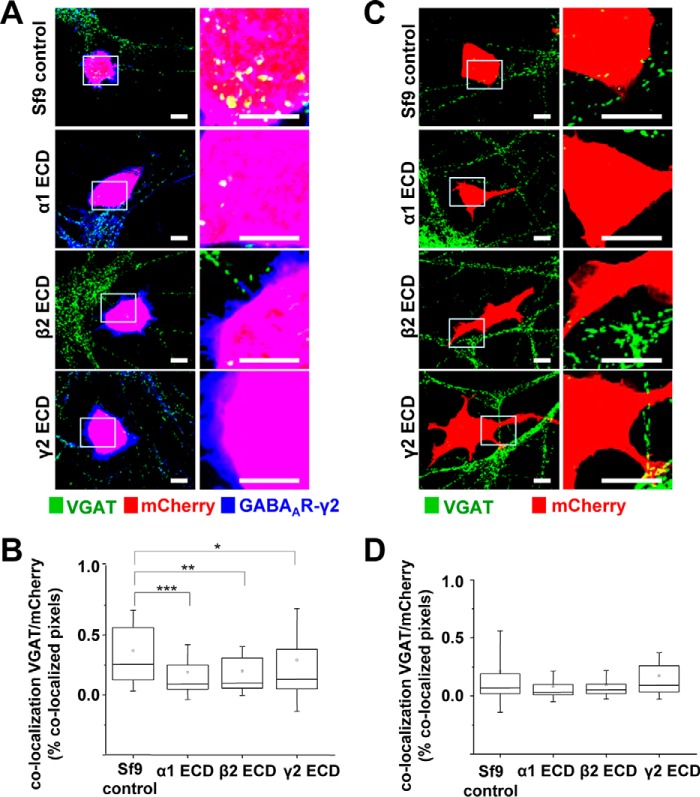
**Inhibition of synaptic contact formation between MSNs and α1β2γ2-HEK293 cells in the presence of purified α1, β2, or γ2 N-terminal ECDs.** Immunolabeling is shown of synaptic contacts between GABAergic presynaptic terminals of MSNs expressing VGAT and α1β2γ2-HEK293 cells (*A*) or control HEK293 cells (*C*) in the presence of control Sf9 cell extracts (*top panel*) or purified α1 (*upper middle panel*), β2 (*lower middle panel*), or γ2 N-terminal ECD (*bottom panel*). GABAergic terminals were labeled with an anti-VGAT antibody (in *green*); HEK293 cells were labeled with mCherry (in *red*), and the surface-expressed GABA_A_Rs were labeled with a γ2 subunit-specific antibody (in *blue*). A selected area in each image (*left column, white box*) was magnified four times, and these images were included in the *right column. Scale bar,* 10 μm. Quantification of contacts is shown between MSNs and α1β2γ2-HEK293 cells (*B*) or control (*D*) HEK293 cells in the presence of control Sf9 cell extracts or purified α1 (*upper middle panel*), β2 (*lower middle panel*), or γ2 N-terminal ECD (*bottom panel*), expressed as a percentage of co-localized pixels between VGAT-positive MSN terminals and mCherry-expressing HEK293 cells. The data were initially analyzed using the Shapiro-Wilk and Kolmogorov-Smirnov test and subsequently using the non-parametric Mann-Whitney test with a confidence interval of 95%. The *box plots* display the median and IQR; *small squares* represent the mean, and *whiskers* represent the data range within 1 S.D. of the median (α1β2γ2-HEK293: *n* = 44–46 cells from *n* = 3 independent experiments; HEK293: *n* = 23–41 cells from *n* = 3–5 independent experiments). *, *p* < 0.05; **, *p* < 0.01; ***, *p* < 0.001.

**FIGURE 10.**
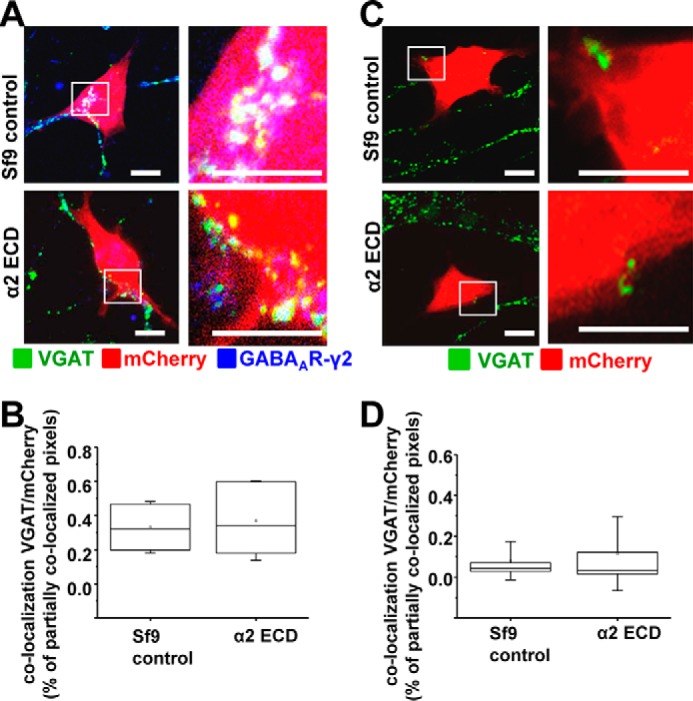
**Purified N-terminal ECD of the α2 subunit does not inhibit the formation of synaptic contacts between MSNs and α1β2γ2-HEK293 cells.** Immunolabeling is shown of synaptic contacts between GABAergic presynaptic terminals of MSNs expressing VGAT and α1β2γ2-HEK293 cells (*A*) or control HEK293 cells (*C*) in the presence of control Sf9 cell extracts (*top panel*) or purified α2 N-terminal ECD (*lower panel*). GABAergic terminals were labeled with an anti-VGAT antibody (in *green*); HEK293 cells were labeled with mCherry (in *red*), and the surface expressed GABA_A_Rs were labeled with a γ2 subunit-specific antibody (in *blue*). A selected area in each image (*left column, white box*) was magnified four times, and these images were included in the *right column. Scale bar,* 10 μm. Quantification of contacts is shown between MSNs and α1β2γ2-HEK293 cells (*B*) or control HEK293 cells (*D*) in the presence of control Sf9 cell extracts or purified α2 N-terminal ECD, expressed as a percentage of co-localized pixels between VGAT-positive MSN terminals and mCherry-expressing HEK293 cells. The data were initially analyzed using the Shapiro-Wilk and Kolmogorov-Smirnov test and subsequently using the non-parametric Mann-Whitney test with a confidence interval of 95%. The *box plots* display the median and IQR; *small squares* represent the mean, and *whiskers* represent the data range within 1 S.D. of the median (α1β2γ2-HEK293: *n* = 18 cells from *n* = 2 independent experiments; HEK293: *n* = 17 cells from *n* = 2 independent experiments).

To elucidate whether the synaptogenic effects of GABA_A_Rs were directly mediated by their N-terminal ECDs, the purified α1, α2, β2, and γ2 ECDs were each separately applied 30 min after plating the mCherry-transfected α1β2γ2-HEK293 into the MSN cultures. In controls, the extract from untransfected Sf9 cells, which was prepared in parallel with the GABA_A_R N-terminal ECDs using the same purifications steps, was added to the α1β2γ2-HEK293 and MSN co-cultures. The cells were fixed after 24 h, and stained using the VGAT and γ2-specific antibodies, and formation and quantification of synaptic contacts were carried out as described above (Figs. 9 and 10). A significant reduction in the number of synaptic contacts between the α1β2γ2-HEK293 and MSNs was observed in the presence of each of the exogenous N-terminal ECDs: α1, β2 or γ2 (Fig. 9*A*). ImageJ analysis revealed that co-localization between VGAT-positive presynaptic terminals and mCherry-expressing α1β2γ2-HEK293 cells was significantly reduced compared with Sf9 control extract, with a median of 0.09% IQR = 0.05–0.26 (*n* = 45 cells; *p* < 0.001), 0.10% IQR = 0.05–0.32 (*n* = 44 cells; *p* < 0.01), and 0.13% IQR = 0.05–0.38 (*n* = 46 cells; *p* < 0.05), respectively, compared with the median of 0.25% IQR = 0.12–0.57 in controls (*n* = 46 cells; all from *n* = 3 independent experiments; Fig. 9*B*). However, addition of the exogenous α2 N-terminal ECD had no effect on synapse formation in these co-cultures (Sf9 cell control extracts: 0.32% IQR = 0.19–0.47, *n* = 17 cells, *versus* α2 ECD: 0.34% IQR = 0.17–0.61, *n* = 18 cells; both from *n* = 2 independent experiments; *p* > 0.05; Fig 10*A*).

Consistent with the control experiments described above (Figs. 1, 3, 5, and 6, *C* and *D*), no synaptic contact formation was observed between mCherry-HEK293 cells that did not express GABA_A_Rs and MSNs, following the application of purified α1, β2 or γ2 N-terminal ECDs or the control Sf9 extract (Fig. 9*C*). Results from ImageJ analysis confirmed this observation revealing that co-localization between VGAT-positive terminals and mCherry-expressing HEK293 cells was minimal in all four conditions (Sf9 control extracts: 0.08% IQR = 0.03–0.19; *n* = 40 cells, *versus* α1 ECD: 0.04% IQR = 0.02–0.11; *n* = 23 cells, *versus* β2 ECD 0.05% IQR = 0.02–0.12), *n* = 23 cells, *versus* γ2 ECD: 0.09% IQR = 0.04–0.28), *n* = 41 cells; from *n* = 3 to 5 independent experiments; Fig. 9*D*). Similar results were obtained after application of the α2 ECD (Sf9 control extracts: 0.04% IQR = 0.03–0.08, *n* = 16 cells, *versus* α2 ECD: 0.03% IQR = 0.01–0.13; *n* = 16 cells, both from *n* = 2 independent experiments; *p* > 0.05; Fig 10*B*).

These experiments indicate that the N-terminal extracellular domains of each of the subunits incorporated into the GABA_A_R pentamer (α, β, or γ) contribute to synaptic contact formation, and they suggest that multiple protein-protein interactions between these domains and proteins residing on the surface of GABAergic presynaptic terminals and/or within the synaptic matrix may be the key mediators in the assembly of GABAergic synapses.

## Discussion

Delineating the molecular mechanisms that regulate development of the central nervous system and the formation of synaptic connections between neurons with the precision required for execution of complex behaviors, cognition, and learning is of fundamental importance in neurobiology. One of the key questions remaining to be answered is how the initiation of synaptic connections between neurons actually occurs. Because of a large number of proteins postulated to mediate these processes and the sheer complexity of their possible molecular interactions, exactly how these complex inter-neuronal connections are formed is often studied using reduced *in vitro* co-culture systems. Although far from the situation *in vivo* and subject to all the caveats that should surround any study in a reduced system, this approach has allowed us to establish that GABA_A_Rs have the ability to initiate the adhesion of GABAergic nerve terminals and formation of structurally and functionally competent GABAergic inhibitory synapses (33, 53). These *in vitro* findings are supported by the *in vivo* evidence from GABA_A_ α1 and α2 subunit knock-out mice demonstrating that the lack of these subunits in the hippocampus leads to prominent structural changes in specific types of inhibitory synapses (54). In addition, *in vitro* co-culture assays have been used extensively to characterize a number of other synaptogenic proteins (27–31).

GABA_A_Rs are structurally diverse and can be assembled from as many as 16 different subunits that exhibit specific patterns of expression within different brain regions (2). Based on structural and functional diversity of these receptors, we have hypothesized that GABA_A_Rs can initiate the formation of specific types of GABAergic inhibitory synapses in which the functional properties of both the presynaptic inhibitory neurons and the GABA_A_R subtypes present postsynaptically are closely matched to allow efficient transmission of signals (55).

To characterize the structural basis for synaptogenic activity of GABA_A_Rs and to test the latter hypothesis, in this study we have investigated how the subunit composition of GABA_A_Rs affects their ability to initiate synaptic contact formation, and we have demonstrated that the N-terminal extracellular domain of each of the subunits incorporated into the GABA_A_R pentamer directly participates in this process. Thus, engineering the HEK293 cells to stably express the α1β2-, α1β3-, α2β2-, or α2β3-GABA_A_Rs, in the presence or absence of the γ2 subunit, allowed us to examine not only whether the presence of the γ2 subunit was necessary for contact formation, but also whether the subtype of α or β subunit present in the pentameric receptor can influence its ability to promote synaptic contact formation with GABAergic MSNs in co-culture. The surface expression of GABA_A_Rs in these cell lines was assessed by immunolabeling of PFA-fixed cells with antibodies specific for the extracellular epitopes of these receptors. Although the labeling appeared not to delineate the plasma membrane as precisely as expected, likely due to partial permeabilization with PFA, surface expression of GABA_A_Rs was further demonstrated in electrophysiological experiments by detection of GABA_A_R-mediated currents evoked by bath-applied GABA (Figs. 2, *C* and *D*, and 4, *C* and *D*).

Quantification of synaptic contacts labeled with VGAT or FM4-64FX dye has clearly indicated that the presence of the γ2 subunit was necessary but not sufficient for the formation of synaptic contacts in almost all combinations tested apart from the α2β3γ2 combination, which showed no activity at all, and it was similar to the control HEK293 cells that did not express any GABA_A_Rs. These findings are in agreement with the previous experimental evidence demonstrating that the γ2 subunit is almost uniformly present in all GABAergic synapses in the CNS (56) and that γ2 subunit knockouts exhibit a loss of GABA_A_R clusters and a reduction in GABA_A_Rs-mediated synaptic function both during synaptogenesis and at mature synapses (9, 14, 19–23).

It is of interest to note that in our immunolabeling experiments there was little evidence for clustering of γ2-containing GABA_A_Rs at synaptic contacts, which could be explained by an already very high level of these receptors at the cell surface. Moreover, GABA_A_R clustering protein gephyrin was not detected in our cell lines using immunolabeling with specific antibodies. That gephyrin, if present, is not necessary for synaptic contact formation in this system is indicated by the lack of innervation of control HEK293 cells or α/β-HEK293 cells by MSN axons. Another protein reported to play an important role in postsynaptic clustering of GABA_A_Rs in neurons is collybistin, but this protein was reported to lack synaptogenic activity (57) and is not expressed in HEK293 cells (58).

In our experiments, innervation of HEK293 cells expressing a specific combination of GABA_A_R subunits was directly compared with that of control HEK293 cells, which did not express any GABA_A_Rs, as these were always plated onto coverslips containing the same preparation of MSNs. Therefore, the differences in innervation observed between different subunit combinations can only be compared indirectly by comparing the fold increase in contact formation of these normalized to control HEK293 cells. These comparisons have indicated that the presence of a specific α/β subunit combination within the receptor pentamer also influences the degree of innervation of these cells by MSN axons. For example, the α1β2γ2 combination showed the greatest ability to promote contact formation (∼7-fold increase): the α2β2γ2-combination evoked an ∼5-fold increase, and the α2β3γ2 combination failed to promote contact formation significantly compared, that is, with their respective controls. Importantly, these apparent differences in the ability of different GABA_A_R subunit combinations to promote innervation of HEK293 cells by co-cultured MSNs *in vitro* are in agreement with the expression profiles of GABA_A_R subunits in neurons that are the predominant synaptic target for MSNs *in vivo*. Thus, axons of striatal MSNs predominantly innervate neurons in the globus pallidus and the substantia nigra pars reticulata, and these neurons most commonly express the α1β2γ2-GABA_A_R combination (24–26). Striatal MSNs themselves predominantly express the α2β3γ2 GABA_A_R subunit combination, but they receive synaptic inputs from striatal GABAergic interneurons much more frequently than from other MSNs (37, 59, 60). Thus, striatal MSNs appear to have preference toward the α1β2γ2-GABA_A_R combination as their synaptic targets.

An important question emerging from these experiments is as follows: How are these different subtypes of GABA_A_Rs recognized by GABAergic inputs during the initiation of synaptic contacts? As the large N-terminal ECDs of the GABA_A_R subunits reside within the synaptic cleft, it is possible that these domains bind directly to some yet to be identified presynaptic protein(s) and that these interactions could initiate contact formation and hold the pre- and postsynaptic membranes in close proximity, while the other synaptic adhesion protein interactions develop. Although many other synaptic adhesion proteins also take part in synapse formation and consolidation, the interactions mediated by GABA_A_R ECDs may be required to ensure that the preferred postsynaptic receptor subtypes for a given presynaptic input are selected. To determine whether the synaptogenic effects of the GABA_A_R subunits were directly mediated by their N-terminal ECDs, purified α1, β2, and γ2 ECDs were added to the co-cultures of α1β2γ2-HEK293 cells and MSNs immediately after plating. Application of exogenous ECDs of the abovementioned subunits, but not of the α2 subunit ECD used as a negative control, inhibited contact formation between the MSNs and HEK293 cells, suggesting that synaptogenic effects of GABA_A_Rs are indeed mediated, at least in part, by their ECDs. The specificity of purified ECDs demonstrated in these experiments strongly suggests that their conformation is similar to the confirmation of the endogenous ECDs but also that protein-protein interactions occurring during synaptic contact formation are indeed specific for the subunits incorporated into the GABA_A_Rs. These findings are in agreement with the results demonstrating that inhibition of GABA_A_R activity by a competitive antagonist bicuculline had no significant effect on synaptic contact formation in these co-cultures. The exact mechanism by which the applied ECDs inhibit contact formation remains to be determined and will require further experimentation.

Direct *in vivo* evidence for the role for GABA_A_Rs in synapse assembly has yet to emerge. The challenge lies in the complexity and multiplicity of GABA_A_R subtypes (1, 2) as well as other pre- and post-synaptic proteins found to populate the synaptic cleft. For example, a prominent synaptogenic activity of presynaptic neurexins and their postsynaptic partners neuroligins has been demonstrated *in vitro* by a number of studies (27–31) but also questioned by the results from the *in vivo* analysis of GABAergic synapses in neuroligin knock-out mice, which showed that synapse formation was intact (61). However, prominent impairments in inhibitory synaptic transmission observed in these mice have led to a conclusion that neuroligins are important for functional maturation rather than initiation of synapses. A possible explanation for these results, as well as for our findings in co-culture systems with GABA_A_R-HEK293 cell lines that do not express neuroligins (33), is that different subtypes of GABA_A_R, via their ECDs, may directly interact with specific isoforms of neurexins, among other presynaptic proteins, as suggested by the *in vitro* binding assays (32).

The contrasting evidence for the importance of neuroligins in synapse formation between our studies and previous co-culture studies (29) could be explained by the different combinations of neuronal cell types and postsynaptic GABA_A_R subtypes tested. Moreover, the high level and consistency of cell surface expression of GABA_A_Rs in HEK293 cell lines employed in our studies, in contrast to the transiently expressed GABA_A_Rs in previous studies, may have been crucial for the reliable detection of synaptic contacts. However, in agreement with the other studies (62), our previously published observations have demonstrated that the number of functional contacts was enhanced significantly by concomitant overexpression of NL2 (33).

In conclusion, the co-culture experiments described here have demonstrated that synaptogenic effects of GABA_A_Rs depend on their individual subunit composition, with the N-terminal ECDs participating directly in the initiation of contacts between the pre- and postsynaptic elements.

## Author Contributions

L. E. B. performed the experiments, analyzed the data, and prepared the figures. A. M. performed the electrophysiological experiments. M. W. N. produced the α1β3 and α2β3-HEK293 cell lines. J. E. A. and L. E. B. optimized the expression and purification of GABA_A_R ECDs. J. N. J. and A. M. T. supervised the experiments. J. N. J. and L. E. B. wrote the manuscript.

## Footnotes

3 The abbreviations used are:

GABA_A_R: GABA_A_ receptor
MSN: medium spiny neuron
ECD: extracellular domain
VGAT: vesicular GABA transporter
IQR: interquartile range
DIC: differential interference contrast
PFA: paraformaldehyde
EGFP: enhanced GFP
sIPSC: spontaneous inhibitory synaptic current
DIV: days *in vitro*.

## References

[1] Schofield P. R., Darlison M. G., Fujita N., Burt D. R., Stephenson F. A., Rodriguez H., Rhee L. M., Ramachandran J., Reale V., and Glencorse T. A. (1987) Sequence and functional expression of the GABA A receptor shows a ligand-gated receptor super-family. Nature 328, 221–227 3037384 10.1038/328221a0

[2] Sieghart W. (2006) Structure, pharmacology, and function of GABAA receptor subtypes. Adv. Pharmacol. 54, 231–263 17175817 10.1016/s1054-3589(06)54010-4

[3] Möhler H. (2006) GABA(A) receptor diversity and pharmacology. Cell Tissue Res. 326, 505–516 16937111 10.1007/s00441-006-0284-3

[4] Miller P. S., and Aricescu A. R. (2014) Crystal structure of a human GABAA receptor. Nature 512, 270–275 24909990 10.1038/nature13293 PMC4167603

[5] Deidda G., Bozarth I. F., and Cancedda L. (2014) Modulation of GABAergic transmission in development and neurodevelopmental disorders: investigating physiology and pathology to gain therapeutic perspectives. Front. Cell. Neurosci. 8, 119 24904277 10.3389/fncel.2014.00119 PMC4033255

[6] Braat S., and Kooy R. F. (2015) The GABAA receptor as a therapeutic target for neurodevelopmental disorders. Neuron 86, 1119–1130 26050032 10.1016/j.neuron.2015.03.042

[7] Nuss P. (2015) Anxiety disorders and GABA neurotransmission: a disturbance of modulation. Neuropsychiatr. Dis. Treat. 11, 165–175 25653526 10.2147/NDT.S58841 PMC4303399

[8] Unwin N. (1993) Neurotransmitter action: opening of ligand-gated ion channels. Cell 72, 31–41 7679054 10.1016/s0092-8674(05)80026-1

[9] Günther U., Benson J., Benke D., Fritschy J. M., Reyes G., Knoflach F., Crestani F., Aguzzi A., Arigoni M., and Lang Y. (1995) Benzodiazepine-insensitive mice generated by targeted disruption of the γ2 subunit gene of γ-aminobutyric acid type A receptors. Proc. Natl. Acad. Sci. U.S.A. 92, 7749–7753 7644489 10.1073/pnas.92.17.7749 PMC41223

[10] Homanics G. E., DeLorey T. M., Firestone L. L., Quinlan J. J., Handforth A., Harrison N. L., Krasowski M. D., Rick C. E., Korpi E. R., Mäkelä R., Brilliant M. H., Hagiwara N., Ferguson C., Snyder K., and Olsen R. W. (1997) Mice devoid of γ-aminobutyrate type A receptor β3 subunit have epilepsy, cleft palate, and hypersensitive behavior. Proc. Natl. Acad. Sci. U.S.A. 94, 4143–4148 9108119 10.1073/pnas.94.8.4143 PMC20582

[11] Rudolph U., Crestani F., Benke D., Brünig I., Benson J. A., Fritschy J. M., Martin J. R., Bluethmann H., and Möhler H. (1999) Benzodiazepine actions mediated by specific γ-aminobutyric acid(A) receptor subtypes. Nature 401, 796–800 10548105 10.1038/44579

[12] Löw K., Crestani F., Keist R., Benke D., Brünig I., Benson J. A., Fritschy J. M., Rülicke T., Bluethmann H., Möhler H., and Rudolph U. (2000) Molecular and neuronal substrate for the selective attenuation of anxiety. Science 290, 131–134 11021797 10.1126/science.290.5489.131

[13] Crestani F., and Rudolph U. (2015) Behavioral functions of GABAA receptor subtypes–the Zurich experience. Adv. Pharmacol. 72, 37–51 25600366 10.1016/bs.apha.2014.10.001

[14] Essrich C., Lorez M., Benson J. A., Fritschy J. M., and Lüscher B. (1998) Postsynaptic clustering of major GABAA receptor subtypes requires the γ2 subunit and gephyrin. Nat. Neurosci. 1, 563–571 10196563 10.1038/2798

[15] Schweizer C., Balsiger S., Bluethmann H., Mansuy I. M., Fritschy J. M., Mohler H., and Lüscher B. (2003) The γ2 subunit of GABA(A) receptors is required for maintenance of receptors at mature synapses. Mol. Cell. Neurosci. 24, 442–450 14572465 10.1016/s1044-7431(03)00202-1

[16] Connolly C. N., Wooltorton J. R., Smart T. G., and Moss S. J. (1996) Subcellular localization of γ-aminobutyric acid type A receptors is determined by receptor β subunits. Proc. Natl. Acad. Sci. U.S.A. 93, 9899–9904 8790428 10.1073/pnas.93.18.9899 PMC38526

[17] Connolly C. N., Krishek B. J., McDonald B. J., Smart T. G., and Moss S. J. (1996) Assembly and cell surface expression of heteromeric and homomeric γ-aminobutyric acid type A receptors. J. Biol. Chem. 271, 89–96 8550630 10.1074/jbc.271.1.89

[18] Klausberger T., Roberts J. D., and Somogyi P. (2002) Cell type- and input-specific differences in the number and subtypes of synaptic GABA(A) receptors in the hippocampus. J. Neurosci. 22, 2513–2521 11923416 10.1523/JNEUROSCI.22-07-02513.2002 PMC6758298

[19] Thomson A. M., and Jovanovic J. N. (2010) Mechanisms underlying synapse-specific clustering of GABA(A) receptors. Eur. J. Neurosci. 31, 2193–2203 20550567 10.1111/j.1460-9568.2010.07252.x

[20] Thomson A. M., Bannister A. P., Hughes D. I., and Pawelzik H. (2000) Differential sensitivity to Zolpidem of IPSPs activated by morphologically identified CA1 interneurons in slices of rat hippocampus. Eur. J. Neurosci. 12, 425–436 10712623 10.1046/j.1460-9568.2000.00915.x

[21] Nyíri G., Freund T. F., and Somogyi P. (2001) Input-dependent synaptic targeting of α(2)-subunit-containing GABA(A) receptors in synapses of hippocampal pyramidal cells of the rat. Eur. J. Neurosci. 13, 428–442 11168550 10.1046/j.1460-9568.2001.01407.x

[22] Ali A. B., and Thomson A. M. (2008) Synaptic α5 subunit-containing GABAA receptors mediate IPSPs elicited by dendrite-preferring cells in rat neocortex. Cereb. Cortex 18, 1260–1271 17951598 10.1093/cercor/bhm160

[23] Shen K., and Scheiffele P. (2010) Genetics and cell biology of building specific synaptic connectivity. Annu. Rev. Neurosci. 33, 473–507 20367446 10.1146/annurev.neuro.051508.135302 PMC3082953

[24] Siddiqui T. J., and Craig A. M. (2011) Synaptic organizing complexes. Curr. Opin. Neurobiol. 21, 132–143 20832286 10.1016/j.conb.2010.08.016 PMC3016466

[25] Kubota Y. (2014) Untangling GABAergic wiring in the cortical microcircuit. Curr. Opin. Neurobiol. 26, 7–14 24650498 10.1016/j.conb.2013.10.003

[26] Fritschy J. M., Panzanelli P., and Tyagarajan S. K. (2012) Molecular and functional heterogeneity of GABAergic synapses. Cell. Mol. Life Sci. 69, 2485–2499 22314501 10.1007/s00018-012-0926-4 PMC11115047

[27] Scheiffele P., Fan J., Choih J., Fetter R., and Serafini T. (2000) Neuroligin expressed in nonneuronal cells triggers presynaptic development in contacting axons. Cell 101, 657–669 10892652 10.1016/s0092-8674(00)80877-6

[28] Graf E. R., Zhang X., Jin S. X., Linhoff M. W., and Craig A. M. (2004) Neurexins induce differentiation of GABA and glutamate postsynaptic specializations via neuroligins. Cell 119, 1013–1026 15620359 10.1016/j.cell.2004.11.035 PMC2826211

[29] Dong N., Qi J., and Chen G. (2007) Molecular reconstitution of functional GABAergic synapses with expression of neuroligin-2 and GABAA receptors. Mol. Cell. Neurosci. 35, 14–23 17336090 10.1016/j.mcn.2007.01.013

[30] Krueger D. D., Tuffy L. P., Papadopoulos T., and Brose N. (2012) The role of neurexins and neuroligins in the formation, maturation, and function of vertebrate synapses. Curr. Opin. Neurobiol. 22, 412–422 22424845 10.1016/j.conb.2012.02.012

[31] Tanaka H., Miyazaki N., Matoba K., Nogi T., Iwasaki K., and Takagi J. (2012) Higher-order architecture of cell adhesion mediated by polymorphic synaptic adhesion molecules neurexin and neuroligin. Cell Rep. 2, 101–110 22840401 10.1016/j.celrep.2012.06.009

[32] Zhang C., Atasoy D., Araç D., Yang X., Fucillo M. V., Robison A. J., Ko J., Brunger A. T., and Südhof T. C. (2010) Neurexins physically and functionally interact with GABA(A) receptors. Neuron 66, 403–416 20471353 10.1016/j.neuron.2010.04.008 PMC3243752

[33] Fuchs C., Abitbol K., Burden J. J., Mercer A., Brown L., Iball J., Anne Stephenson F., Thomson A. M., and Jovanovic J. N. (2013) GABA(A) receptors can initiate the formation of functional inhibitory GABAergic synapses. Eur. J. Neurosci. 38, 3146–3158 23909897 10.1111/ejn.12331 PMC4028986

[34] Fritschy J. M., and Mohler H. (1995) GABAA-receptor heterogeneity in the adult rat brain: differential regional and cellular distribution of seven major subunits. J. Comp. Neurol. 359, 154–194 8557845 10.1002/cne.903590111

[35] Gross A., Sims R. E., Swinny J. D., Sieghart W., Bolam J. P., and Stanford I. M. (2011) Differential localization of GABA(A) receptor subunits in relation to rat striatopallidal and pallidopallidal synapses. Eur. J. Neurosci. 33, 868–878 21219474 10.1111/j.1460-9568.2010.07552.x

[36] Fujiyama F., Fritschy J. M., Stephenson F. A., and Bolam J. P. (2000) Synaptic localization of GABA(A) receptor subunits in the striatum of the rat. J. Comp. Neurol. 416, 158–172 10581463

[37] Tepper J. M., and Bolam J. P. (2004) Functional diversity and specificity of neostriatal interneurons. Curr. Opin. Neurobiol. 14, 685–692 15582369 10.1016/j.conb.2004.10.003

[38] Nelson A. B., and Kreitzer A. C. (2014) Reassessing models of basal ganglia function and dysfunction. Annu. Rev. Neurosci. 37, 117–135 25032493 10.1146/annurev-neuro-071013-013916 PMC4416475

[39] Obeso J. A., Rodriguez-Oroz M. C., Stamelou M., Bhatia K. P., and Burn D. J. (2014) The expanding universe of disorders of the basal ganglia. Lancet 384, 523–531 24954674 10.1016/S0140-6736(13)62418-6

[40] Silberberg G., and Bolam J. P. (2015) Local and afferent synaptic pathways in the striatal microcircuitry. Curr. Opin. Neurobiol. 33, 182–187 26051382 10.1016/j.conb.2015.05.002

[41] Sims R. E., Woodhall G. L., Wilson C. L., and Stanford I. M. (2008) Functional characterization of GABAergic pallidopallidal and striatopallidal synapses in the rat globus pallidus *in vitro*. Eur. J. Neurosci. 28, 2401–2408 19087170 10.1111/j.1460-9568.2008.06546.x

[42] Banker G., and Goslin K. (1988) Developments in neuronal cell culture. Nature 336, 185–186 3185736 10.1038/336185a0

[43] Goffin D., Ali A. B., Rampersaud N., Harkavyi A., Fuchs C., Whitton P. S., Nairn A. C., and Jovanovic J. N. (2010) Dopamine-dependent tuning of striatal inhibitory synaptogenesis. J. Neurosci. 30, 2935–2950 20181591 10.1523/JNEUROSCI.4411-09.2010 PMC3930856

[44] Jovanovic J. N., Thomas P., Kittler J. T., Smart T. G., and Moss S. J. (2004) Brain-derived neurotrophic factor modulates fast synaptic inhibition by regulating GABA(A) receptor phosphorylation, activity, and cell-surface stability. J. Neurosci. 24, 522–530 14724252 10.1523/JNEUROSCI.3606-03.2004 PMC6729993

[45] Porcher C., Hatchett C., Longbottom R. E., McAinch K., Sihra T. S., Moss S. J., Thomson A. M., and Jovanovic J. N. (2011) Positive feedback regulation between γ-aminobutyric acid type A (GABA(A)) receptor signaling and brain-derived neurotrophic factor (BDNF) release in developing neurons. J. Biol. Chem. 286, 21667–21677 21474450 10.1074/jbc.M110.201582 PMC3122223

[46] Duggan M. J., and Stephenson F. A. (1990) Biochemical evidence for the existence of γ-aminobutyrateA receptor iso-oligomers. J. Biol. Chem. 265, 3831–3835 2154490

[47] Tretter V., Ehya N., Fuchs K., and Sieghart W. (1997) Stoichiometry and assembly of a recombinant GABAA receptor subtype. J. Neurosci. 17, 2728–2737 9092594 10.1523/JNEUROSCI.17-08-02728.1997 PMC6573102

[48] Mhatre M. C., Pena G., Sieghart W., and Ticku M. K. (1993) Antibodies specific for GABAA receptor α subunits reveal that chronic alcohol treatment down-regulates α-subunit expression in rat brain regions. J. Neurochem. 61, 1620–1625 8228981 10.1111/j.1471-4159.1993.tb09795.x

[49] Chai H., Vasudevan S. G., Porter A. G., Chua K. L., Oh S., and Yap M. (1993) Glycosylation and high-level secretion of human tumour necrosis factor-β in recombinant baculovirus-infected insect cells. Biotechnol. Appl. Biochem. 18, 259–273 8297505

[50] Possee R. D. (1997) Baculoviruses as expression vectors. Curr. Opin. Biotechnol. 8, 569–572 9353228 10.1016/s0958-1669(97)80030-4

[51] Jarvis D. L., and Summers M. D. (1989) Glycosylation and secretion of human tissue plasminogen activator in recombinant baculovirus-infected insect cells. Mol. Cell. Biol. 9, 214–223 2494430 10.1128/mcb.9.1.214-223.1989 PMC362163

[52] Jarvis D. L., Oker-Blom C., and Summers M. D. (1990) Role of glycosylation in the transport of recombinant glycoproteins through the secretory pathway of lepidopteran insect cells. J. Cell. Biochem. 42, 181–191 2341487 10.1002/jcb.240420402

[53] Brown L. E., Fuchs C., Nicholson M. W., Stephenson F. A., Thomson A. M., and Jovanovic J. N. (2014) Inhibitory synapse formation in a co-culture model incorporating GABAergic medium spiny neurons and HEK293 cells stably expressing GABAA receptors. J. Vis. Exp. 93, e52115 25489750 10.3791/52115 PMC4354098

[54] Panzanelli P., Gunn B. G., Schlatter M. C., Benke D., Tyagarajan S. K., Scheiffele P., Belelli D., Lambert J. J., Rudolph U., and Fritschy J. M. (2011) Distinct mechanisms regulate GABAA receptor and gephyrin clustering at perisomatic and axo-axonic synapses on CA1 pyramidal cells. J. Physiol. 589, 4959–4980 21825022 10.1113/jphysiol.2011.216028 PMC3224886

[55] Klausberger T., and Somogyi P. (2008) Neuronal diversity and temporal dynamics: the unity of hippocampal circuit operations. Science 321, 53–57 18599766 10.1126/science.1149381 PMC4487503

[56] Somogyi P., Fritschy J. M., Benke D., Roberts J. D., and Sieghart W. (1996) The γ2 subunit of the GABAA receptor is concentrated in synaptic junctions containing the α1 and β2/3 subunits in hippocampus, cerebellum and globus pallidus. Neuropharmacology 35, 1425–1444 9014159 10.1016/s0028-3908(96)00086-x

[57] Chiou T. T., Bonhomme B., Jin H., Miralles C. P., Xiao H., Fu Z., Harvey R. J., Harvey K., Vicini S., and De Blas A. L. (2011) Differential regulation of the postsynaptic clustering of γ-aminobutyric acid type A (GABAA) receptors by collybistin isoforms. J. Biol. Chem. 286, 22456–22468 21540179 10.1074/jbc.M111.236190 PMC3121391

[58] Kins S., Betz H., and Kirsch J. (2000) Collybistin, a newly identified brain-specific GEF, induces submembrane clustering of gephyrin. Nat. Neurosci. 3, 22–29 10607391 10.1038/71096

[59] Mallet N., Le Moine C., Charpier S., and Gonon F. (2005) Feedforward inhibition of projection neurons by fast-spiking GABA interneurons in the rat striatum *in vivo*. J. Neurosci. 25, 3857–3869 15829638 10.1523/JNEUROSCI.5027-04.2005 PMC6724938

[60] Tepper J. M., Abercrombie E. D., and Bolam J. P. (2007) Basal ganglia macrocircuits. Prog. Brain Res. 160, 3–7 17499105 10.1016/S0079-6123(06)60001-0

[61] Zhang B., Chen L. Y., Liu X., Maxeiner S., Lee S. J., Gokce O., and Südhof T. C. (2015) Neuroligins sculpt cerebellar Purkinje-cell circuits by differential control of distinct classes of synapses. Neuron 87, 781–796 26291161 10.1016/j.neuron.2015.07.020 PMC4545494

[62] Fu Z., and Vicini S. (2009) Neuroligin-2 accelerates GABAergic synapse maturation in cerebellar granule cells. Mol. Cell. Neurosci. 42, 45–55 19463950 10.1016/j.mcn.2009.05.004 PMC2741007

